# Genetics of adaptation in modern chicken

**DOI:** 10.1371/journal.pgen.1007989

**Published:** 2019-04-29

**Authors:** Saber Qanbari, Carl-Johan Rubin, Khurram Maqbool, Steffen Weigend, Annett Weigend, Johannes Geibel, Susanne Kerje, Christine Wurmser, Andrew Townsend Peterson, I. Lehr Brisbin, Ruedi Preisinger, Ruedi Fries, Henner Simianer, Leif Andersson

**Affiliations:** 1 Animal Breeding and Genetics Group, Department of Animal Sciences, University of Göttingen, Göttingen, Germany; 2 Department of Animal Biotechnology, Agricultural Biotechnology Research Institute of Iran (ABRII), Agricultural Research, Education and Extension Organization (AREEO), Karaj, Iran; 3 Science for Life Laboratory, Department of Medical Biochemistry and Microbiology, Uppsala University, Uppsala, Sweden; 4 Department of Animal Breeding and Genetics, Swedish University of Agricultural Sciences, Uppsala, Sweden; 5 Friedrich-Loeffler-Institut, Neustadt, Germany; 6 Center for Integrated Breeding Research, University of Göttingen, Göttingen, Germany; 7 Chair of Animal Breeding, Technical University Munich, Freising, Germany; 8 Biodiversity Institute, University of Kansas, Lawrence, Kansas, United States of America; 9 Savannah River Ecology Laboratory, Odum School of Ecology, University of Georgia, Aiken, South Carolina, United States of America; 10 Lohmann Tierzucht GmbH, Cuxhaven, Germany; 11 Department of Veterinary Integrative Biosciences, Texas A&M University, College Station, United States of America; University of Bern, SWITZERLAND

## Abstract

We carried out whole genome resequencing of 127 chicken including red jungle fowl and multiple populations of commercial broilers and layers to perform a systematic screening of adaptive changes in modern chicken (*Gallus gallus domesticus*). We uncovered >21 million high quality SNPs of which 34% are newly detected variants. This panel comprises >115,000 predicted amino-acid altering substitutions as well as 1,100 SNPs predicted to be stop-gain or -loss, several of which reach high frequencies. Signatures of selection were investigated both through analyses of fixation and differentiation to reveal selective sweeps that may have had prominent roles during domestication and breed development. Contrasting wild and domestic chicken we confirmed selection at the *BCO2* and *TSHR* loci and identified 34 putative sweeps co-localized with *ALX1*, *KITLG*, *EPGR*, *IGF1*, *DLK1*, *JPT2*, *CRAMP1*, *and GLI3*, among others. Analysis of enrichment between groups of wild vs. commercials and broilers vs. layers revealed a further panel of candidate genes including *CORIN*, *SKIV2L2* implicated in pigmentation and *LEPR*, *MEGF10* and *SPEF2*, suggestive of production-oriented selection. SNPs with marked allele frequency differences between wild and domestic chicken showed a highly significant deficiency in the proportion of amino-acid altering mutations (P<2.5×10^−6^). The results contribute to the understanding of major genetic changes that took place during the evolution of modern chickens and in poultry breeding.

## Author summary

Domestic chickens (Gallus gallus domesticus) provide a critical resource for animal proteins for human nutrition worldwide. Chickens were primarily domesticated from the red jungle fowl (Gallus gallus gallus), a bird that still runs wild in most of Southeast Asia. Human driven selection during domestication and subsequent specialization into meat type (broilers) and egg layer (layers) birds has left detectable signatures of selection within the genome of modern chicken. In this study, we performed whole genome sequencing of 127 chicken including the red jungle fowl and multiple populations of commercial broilers and layers to perform a systematic screening of adaptive changes in modern chicken. Analysis of selection provided a comprehensive list of several tens of independent loci that are likely to have contributed to domestication or improving production. SNP by SNP comparison of allele frequency between groups of wild and domestic chicken showed a highly significant deficiency of the proportion of amino acid altering mutations. This implies that commercial birds have undergone purifying selection reducing the frequency of deleterious variants.

## Introduction

The modern chicken (*Gallus gallus domesticus*) was domesticated from the red jungle fowl (RJF) [1], but with some contributions from at least one other closely related species, the grey jungle fowl [2]. Domestic chicken segregate into several hundreds of distinct breeds distributed across the world. During the last century, the domestic chicken has been developed into a major food source by adapting the genome to specialized egg laying (layers) and fast-growing meat birds (broilers) whose productivity far exceeds their wild ancestor as well as the domestic chicken present only 100 years ago. Most modern commercial layers produce ~300 eggs in a year while the RJF usually lay a single clutch of 5–9 eggs per year. Modern broilers rapidly reach a body weight of 4–5 kg compared to the ~1 kg weight of an adult RJF male [3]. The commercial broiler and layer suppliers produce more than 70 billion birds annually to meet current worldwide consumer demands of more than 120 million tons of meat and over 1.2 trillion eggs [4].

The increasing productivity has been achieved through intensive directional selection on production traits over several tens of generations in purebred populations of limited effective population size followed by crossbreeding strategies in the generation of production animals. Maximizing yield however, has been at the price of reduced immunity and accompanied by a number of undesirable traits [5]. These negative effects may either be the result of pleiotropy of genes under selection for increased productivity, hitch-hiking of unfavourable alleles with the alleles under selection, or genetic drift. Understanding the nature of adaptive forces acting on the genome of commercial chicken provides insight into the complex relationship between production, disease and genes while opening up new directions for further improvement of this important farm animal, that is essential for global food security.

The domestic chicken is an excellent model to investigate the genetics of adaptation, as it involves transformation of the ancestral red jungle fowl into a domesticated bird. Furthermore, parallel populations of broilers and layers were independently established from earlier multi-purpose populations by several breeding companies selecting for very similar breeding goals during the last hundred years. This scenario allows us to explore if the same alleles are responsible for the selection response in these parallel populations. In this study, we conducted a systematic comparison of genomic sequence variation from multiple populations of broilers and layers, versus each other and versus RJF to identify genes that underwent selection during domestication and the subsequent specialization of domestic chicken into broiler and layer lines. We report the discovery and characterization of over 21 million SNPs, 34% of which were not previously described. Analysis of selection provides a comprehensive list of candidate loci underlying domestication and/or changes in production-relevant traits. We further report a highly significant (P<2.5×10^−6^) deficiency of amino-acid altering mutations among those showing strong genetic differentiation between RJF and commercial birds.

## Results and discussion

### Detecting millions of high-quality SNPs

The bioinformatics analysis using the described criteria detected ∼26.3 million putative SNPs and INDELs. After filtering, over 21 million high-quality bi-allelic SNPs were retained that were either segregating or fixed for a non-reference within a population. The retained variants were distributed in the genome with an average density of 1 SNP every ~50 bases. About 34% of these SNPs (n = 7,146,382) had not been reported before. The number of SNPs detected in each population varied between 7.6 and 17.4 million (Table 1). For the layer lines, the proportion of segregating variants was rather low, with an average of 57% of total variation, while the corresponding average for the broilers was 65%. RJFt alone carries 86% of all detected variants. These results show that layers have lost a considerable amount of the genetic diversity present in their wild ancestor as also indicated by the significantly lower levels of nucleotide diversity (*π*) in LRs (0.15–0.20%) compared with that observed in RJFt (0.40%; Table 1), although the possibility exists that the nucleotide diversity in RJFt is somewhat inflated if multiple subpopulations in northern Thailand was sampled. The low nucleotide diversity of RJFi (0.13%) is presumably due to the fact that this population has been maintained as a small, closed breeding population for many years. The observed reduction in nucleotide diversity in the layer lines is mainly attributed to small number of founders and many generations of mating within closed lines of limited population size, but also partly due to the effect of linked selection.

**Table 1:** Summary statistics for chicken whole genome resequencing.

Population	Code	Sequencing	N	Depth^1^	nSNPs^2^	*π* (%) ^3^
Red jungle fowl (Thailand)	RJFt	Individual	25	11.0	17,422,645	0.40 ± 0.18
Red jungle fowl (India)	RJFi	Individual	10	2.5	9,470,039	0.13 ± 0.07
Broiler A	BRA	Individual	20	11.5	12,355,756	0.34 ± 0.16
Broiler B	BRB	Individual	20	11.9	11,525,631	0.32 ± 0.16
Pooled Broiler D	BRpD	Pooled	25	40.0	10,498,251	-
White layer	WL	Individual	25	8.1	7,638,111	0.15 ± 0.12
Brown layer	BL	Individual	25	7.6	8,812,787	0.20 ± 0.14
Rhode White (layer)	RWp	Pooled	48	30.0	8,614,223	-

^1^Averaged over number of samples when sequenced individually.

^2^Number of polymorphic sites within population.

^3^Average ± standard deviation of nucleotide diversity estimated in 40kb windows.

We detected 115,107 amino acid-altering SNPs of which 17% were predicted by SIFT to be evolutionary intolerant (SIFT scores = 0.00–0.05), 215,810 synonymous variants, 588,491 variants within untranslated regions and 1,100 stop-gain or -loss variants. An unknown fraction of these will have functional consequences.

### Allele frequency spectrum

**Fig 1:**
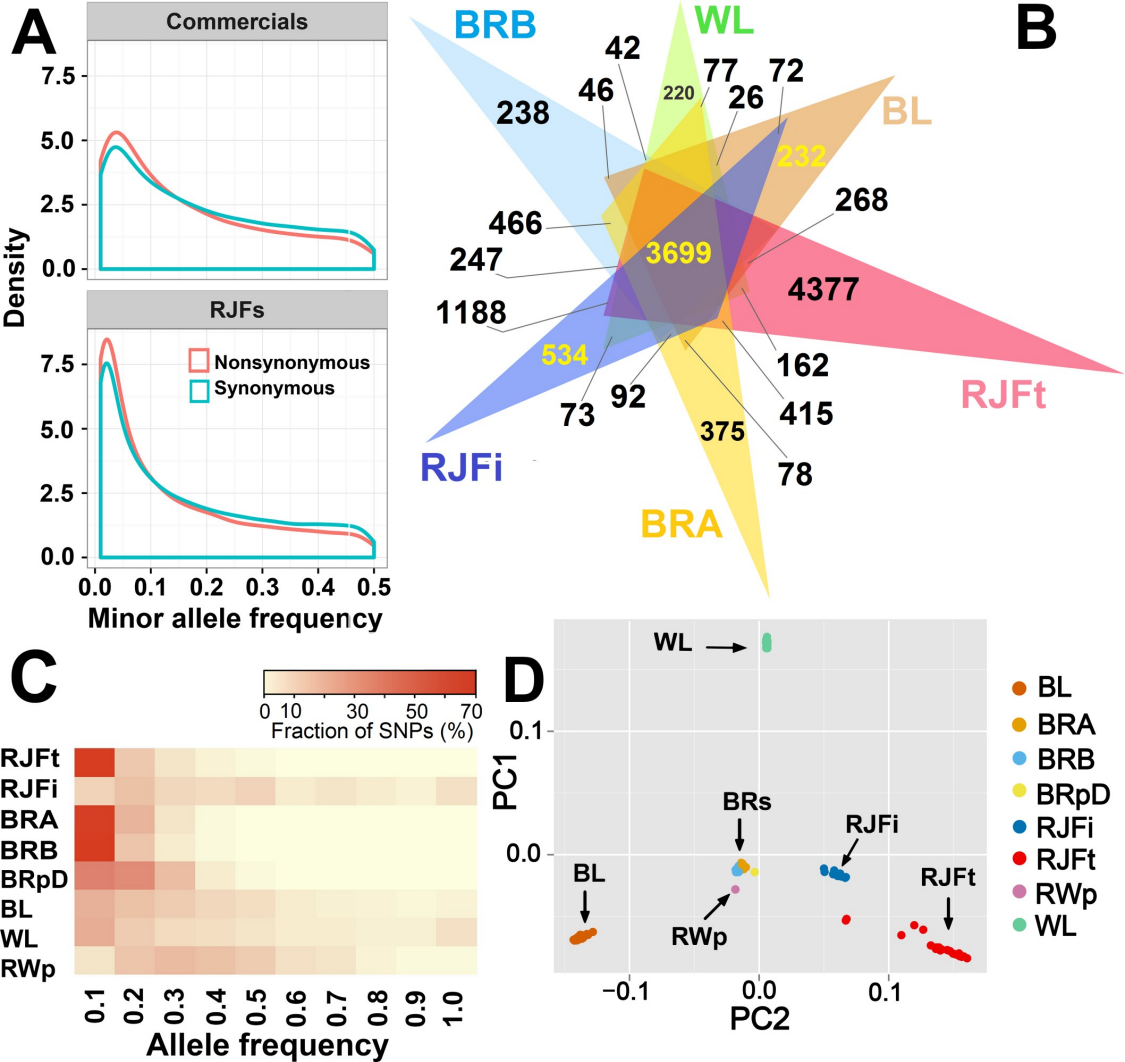
Analysis of SNP diversity. (A) Comparison of the minor allele frequency spectrum of coding sequences in RJFt and commercial populations. (B) Visualization of the distribution of population-specific and group-specific variants detected from individual sequencing only. Each triangle represents the number (10^3^) of variants exclusively segregating or detected in the corresponding population and overlapping sections denote group-specific variants. (C) Heatmap of the allele frequency distribution of population-specific variants. (D) Principal component analysis of chicken populations. Populations are coded as RJFt = red jungle fowl (Thailand), RJFi = red jungle fowl (India), BL = Brown layer, WL = White layer, RWp = Rhode-White pool, BRA = Broiler line A, BRB = Broiler line B and BRpD = Broiler line pool D, BRs = three commercial broiler lines (BRA, BRB and BRpD). show a substantially smaller proportion of rare alleles that can be attributed to the smaller effective population size caused by recent selective breeding leading to loss of rare alleles.

The comparison of the allele frequency profiles of wild and commercial populations reveals substantial differences (Fig 1A; S2 Fig). In wild birds (RJFt), the distribution of allele frequencies shows a marked overrepresentation of infrequent alleles which is consistent with the pattern observed for high-quality data in many other organisms including human and cattle populations [6, 7]. In contrast, commercial populations, particularly layers (S2 Fig), show a substantially smaller proportion of rare alleles that can be attributed to the smaller effective population size caused by recent selective breeding leading to loss of rare alleles. A subtle excess in the proportion of missense relative to synonymous mutations is evident among rare alleles, presumably caused by selection reducing the allele frequency of slightly deleterious mutations [6, 8].

Fig 1B shows the distribution of population- and group-specific variants detected from individual sequencing only. Out of the >18 million variants detected in RJFt, as many as 4.4 million were unique to this population (Fig 1B). This suggests loss of genetic diversity during domestication and breeding, although this might be partly due to genetic differences between the RJF birds used in this study and the ancestral population(s) of red jungle fowl that contributed to chicken domestication. We compared the distributions of population-specific SNPs among commercial and wild chicken to investigate differences in the frequency patterns (Fig 1C). With the exception of the inbred RJFi population, the layer lines exhibit higher frequencies of population-specific alleles. This may be a consequence of a more narrow genetic basis and successive generations of selective breeding in commercial populations to enhance the frequency of favourable alleles. A good proportion of these loci are probably dragged to higher frequencies due to linkage with the target loci under selection [9]. Summary statistics of group-specific variants discovered exclusively in the layer and broiler lines are presented in supplementary Tables 1–4.

**Table 2:** Pairwise *F*_*ST*_ between populations.

	RJFi	BRA	BRB	BL	WL
**RJFt**	0.27 ± 0.12	0.18 ± 0.10	0.20 ± 0.11	0.30 ± 0.14	0.38 ± 0.14
**RJFi**		0.34 ± 0.14	0.37 ± 0.15	0.51 ± 0.19	0.57 ± 0.20
**BRA**			0.09 ± 0.09	0.30 ± 0.16	0.37 ± 0.17
**BRB**				0.32 ± 0.17	0.39 ± 0.18
**BL**					0.53 ± 0.21

Pairwise *F*_*ST*_ values ± (standard deviation) are reported for RJFt = red jungle fowl (Thailand), RJFi = red jungle fowl (India), BL = Brown layer, WL = White layer, RWp, Rhode and White pool, BRA = Broiler line A, BRB = Broiler line B and BRpD = Broiler line pool D.

**Table 3:** List of putative selective sweeps revealed by *F*_*ST*_ analysis.

CHR	BIN_START	BIN_END	nSNPs	*F*_*ST*_	*ZF*_*ST*_	CONTRAST	GENE^a^
1	8600001	8640000	342	0.51	6.28	RJFs/Coms	Gene desert
1	32480001	32520000	647	0.75	7.22	RJFs/LRs,RJFs/Coms	Gene desert
1	55380001	55420000	636	0.77	10.59	RJFs/Coms,RJFs/BRs,RJFs/LRs	*IGFI*
1	55900001	55940000	762	0.59	7.51	RJFs/Coms,RJFs/BRs,RJFs/LRs	*TBXAS1*
1	102160001	102200000	489	0.50	6.10	RJFs/Coms	Gene desert
1	127560001	127600000	388	0.61	6.46	RJFs/BRs,RJFs/Coms	Gene desert
1	142020001	142060000	581	0.50	6.00	RJFs/Coms	Gene desert
2	24680001	24720000	1108	0.55	6.89	RJFs/Coms	*ASNS*, *C1GALT1*
2	25800001	25840000	471	0.68	7.39	RJFs/BRs,RJFs/Coms,BRs/LRs,RJFs/LRs	Gene desert
2	27840001	27880000	248	0.76	6.46	BRs/LRs,RJFs/LRs	Gene desert
2	50840001	50880000	271	0.58	6.04	RJFs/BRs	*GLI3*
2	70840001	70880000	533	0.78	7.63	RJFs/LRs,RJFs/Coms	*HNF4G*
2	73280001	73320000	583	0.59	6.07	RJFs/BRs	*CDH12*
2	82440001	82480000	540	0.51	6.21	RJFs/Coms,RJFs/LRs,RJFs/BRs	Gene desert
2	119220001	119260000	422	0.68	9.15	RJFs/Coms,RJFs/BRs,RJFs/LRs	Gene desert
2	143420001	143460000	459	0.59	7.52	RJFs/Coms,RJFs/LRs	Gene desert
3	29400001	29440000	568	0.86	7.53	BRs/LRs,RJFs/Coms	*GLP1R*
4	56940001	56980000	832	0.52	6.34	RJFs/Coms	*CAMK2D*
4	71740001	71780000	313	0.61	6.34	RJFs/BRs,RJFs/Coms	Gene desert
5	29920001	29960000	604	0.56	7.14	RJFs/Coms,RJFs/BRs,	Gene desert
5	31100001	31140000	512	0.51	6.25	RJFs/Coms,RJFs/BRs,BRs/LRs	Gene desert
5	32300001	32340000	593	0.54	6.68	RJFs/Coms,RJFs/BRs	Gene desert
5	48920001	49400000	355	0.79	6.73	BRs/LRs	*DLK1*
9	12400001	12440000	477	0.55	6.92	RJFs/Coms	*AGTR1*
10	6400001	6440000	836	0.54	6.78	RJFs/Coms,RJFs/LRs	*THSD4*
11	80001	140000	535	0.78	6.60	BRs/LRs,RJFs/LRs	*PLA2G15*,
14	13700001	13740000	1798	0.71	9.65	RJFs/Coms,RJFs/BRs,RJFs/LRs	*JPT2*, *CRAMP1L*
14	14280001	14320000	1877	0.64	8.40	RJFs/Coms,RJFs/BRs,RJFs/LRs	*CCNF*, *gga-mir-1715*
15	2980001	3020000	314	0.71	6.69	RJFs/LRs,RJFs/Coms	*STX2*
24	6140001	6180000	510	0.65	7.00	RJFs/BRs,RJFs/Coms	*BCO2*
26	120001	160000	185	0.63	6.68	RJFs/BRs	*SLC26A8*, *MAPK14*

^a^ All genes residing in the top differentiated windows, some windows carry more than one gene as indicated here.

**Table 4:** A list of candidate genes harbouring missense mutations with ΔAF > 0.7 in two contrasts ‘RJFs vs. Coms’ and ‘BRs vs. LRs’.

				**Frequency of variant allele**^a^														
				Wild		Layers			Broilers									
**Gene**	Chr_Pos	Ref	Alt	RJFt	RJFi	BL	WL	RWp	BRA	BRB	BRpD	ΔRJFsComs	ΔRJFsBRs	ΔRJFsLRs	ΔBRsLRs	AA	SIFT	Function^b^
***GLI3***	2_50876521	T	C	0.06	0.00	1.00	0.00	1.00	0.98	0.95	0.84	**0.76**	0.64	0.89	0.25	K/R	Tolerated_low_confidence (1)	Wing development
***CORIN***	4_67124162	T	A	0.12	0.06	1.00	1.00	1.00	0.85	0.98	0.93	**0.87**	0.91	0.83	0.08	S/T	Tolerated (0.4)	Pigmentation
***KIF7***	10_12832456	G	A	0.06	0.00	0.54	0.88	1.00	0.9	0.8	0.83	**0.79**	0.77	0.82	0.04	V/M	Tolerated (0.17)	Wing development.
***SPEF2***	Z_10819221	G	A	0.03	NA	1.00	0.88	1.00	0.84	1.00	0.21	**0.79**	0.93	0.65	0.28	S/N	Tolerated (0.36)	Chicken feathering
***SKIV2L2***	Z_16761767	C	G	0.71	NA	0.00	0.00	NA	0.00	0.00	0.00	**0.71**	0.71	0.71	0.00	P/A	Tolerated_low_confidence (0.14)	Melanocyte regeneration
***LEPR***	8_28464218	T	C	0.56	NA	0.71	1.00	0.87	0.00	0.07	0.35	0.06	0.30	0.42	**0.72**	C/R	Deleterious (0.01)	Leptin receptor
***IGSF10***	9_23584350	A	C	0.10	NA	0.61	1.00	0.84	0.02	0.07	0.00	0.32	0.72	0.07	**0.78**	I/L	NA	Delayed puberty
***PLEKHM1***	27_1768503	C	G	0.14	NA	0.94	0.84	0.58	0.05	0.00	0.00	0.26	0.65	0.12	**0.77**	G/A	Tolerated (1)	Osteoporosis
***MEGF10***	Z_56413219	T	C	0.20	NA	0.00	0.00	0.00	0.81	0.90	0.78	0.21	0.20	0.63	**0.83**	I/T	Tolerated (0.82)	Muscle generation

^a^Frequencies of variant alleles (Alt) are reported for RJFt = red jungle fowl (Thailand), RJFi = red jungle fowl (India), BL = Brown layer, WL = White layer, RWp, Rhode and White pool, BRA = Broiler line A, BRB = Broiler line B and BRpD = Broiler line pool D. AA = Amino acid substitutions, SIFT<0.05 indicates a likely deleterious missense mutation. Sites with ΔAF>0.7 in two contrasts of ‘RJFs vs. Coms’ and ‘BRs vs. LRs’ are in bold.

### Principal component analysis of genetic relationships

We performed a comprehensive analysis of genetic similarity based on genotypes from >21 million SNPs. As expected, individually sequenced birds from the same population clustered together (Fig 1D; S3 Fig). The white (WL) and brown (BL) laying birds clustered distantly, although they are both layers, a result consistent with previous data [10]. Rhode White (RWp) is a layer breed developed by crossing white and brown layers and is located in the middle of the plot. The clusters of RJFs from Thailand and India were in fairly close proximity to one other. Broilers showed a strong clustering in the middle of the plot, probably due to the common ancestor of all, rooted back to the Cornish breed [11]. These results provide important background information for our attempts to identify loci under selection in the domestic populations.

### Detecting selective sweeps

#### a. Analysis of genetic differentiation

The level of genetic differentiation varies among chromosomes, annotation categories as well as groups of birds (S4 Fig). To detect putative selective sweeps, we first searched the genome for regions with high degrees of differentiation between groups (RJF, LRs and BRs). Across the genome we observed the largest *F*_*ST*_ values in contrasts between populations with the lowest nucleotide diversities reflecting genetic drift (Tables 1 and 2). *F*_*ST*_ values were estimated in sliding 40 kb windows along the genome in steps of 20 kb. The size of a selective sweep depends on multiple factors such as the local recombination rate, selection intensity, and the number of generations that passed from the time when a favourable mutation arose and it became fixed. Thus, selective sweeps vary in size due to several variables, making it difficult to determine an optimal window size in which to search for signatures of selection. Thus, we cannot rule out that our approach may have failed to detect sweeps that would have been detected using other fixed or variable window sizes. The distribution of window-Z*F*_*ST*_ values are plotted in S4C Fig for all comparisons. Since only windows with >10 SNPs were analysed, the number of windows available for analysis varied from 46,146 to 46,150 per comparison (S5–S8 Tables).

The profile of *F*_*ST*_ also varied among comparisons and chromosomes (S4 Fig), which complicates defining a threshold to distinguish true selective sweeps from regions showing genetic differentiation due to genetic drift. We therefore defined putative sweeps as those reaching a Z*F*_*ST*_ score ≥ 6, as these were in the extreme upper end of the distribution (S4C Fig). We however believe that loci further down the list still merit further examination in follow-up studies. All windows with Z*F*_*ST*_ ≥ 4 in any of the comparisons are listed in S9 Table.

**Fig 2:**
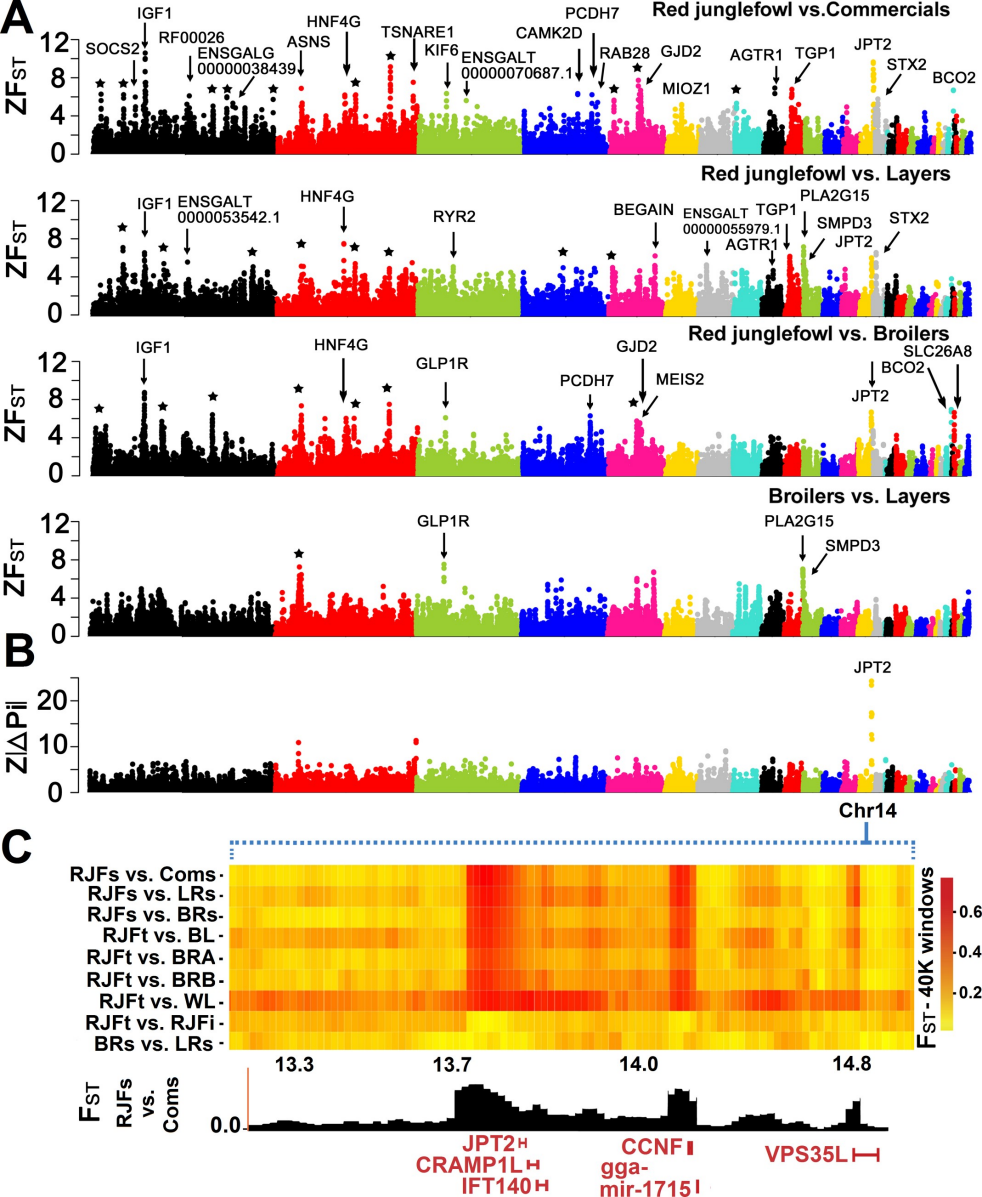
Genome-wide visualization of candidate selective sweeps. Each dot represents a 40 kb window in steps of 20 kb along the genome. (A) *ZF*_*ST*_ scores in different contrasts of chicken populations. Candidate genes are indicated for each signal. Signals marked by a star represent regions lacking annotated genes. (B) Manhattan plot of the *Z*|*ΔPi*| scores between two RJF and four commercial populations. (C) High resolution illustration of putative sweeps on GGA14. The heatmap visualizes the region as *F*_*ST*_ values among multiple populations and groups, where the genes or known elements overlapping the candidate sweeps are indicated underneath.

Only ~0.13% of the windows (n = 60) had a Z*F*_*ST*_ score ≥ 6 in the ‘RJFs vs. Coms’ comparison, and the corresponding fractions were ~0.05% for ‘BRs vs. LRs’ (n = 41), ~0.03% for RJFs vs. LRs (n = 66) and ~0.07% for ‘RJFs vs. BRs’ (n = 90). In total, 31 putative sweeps were mapped with Z*F*_*ST*_-scores exceeding the threshold at least in one of the contrasts (Table 3). We used the yellow skin (*BCO2*) locus [2] and the *TSHR* locus [12] as proofs of principle showing that our approach can reveal established sweeps. We observed an *F*_*ST*_ value of 0.65 (Z*F*_*ST*_ = 7.0) over *BCO2* (Fig 2A) and the localization perfectly overlapped the previously defined sweeps. The window harbouring the *TSHR* locus showed an *F*_*ST*_ value of 0.34 (Z*F*_*ST*_ = 3.4) in the ‘RJFs vs. Coms’ contrast residing within 9% of top differentiated windows (S6 Table). Another signal (Z*F*_*ST*_ = 10.6) overlapping a previously detected sweep was mapped on chromosome 1 over *IGF1*, which encodes insulin-like growth factor 1, an important growth factor associated with body size in dogs [13]. This signal appeared in three out of four contrasts where RJFs were included and were maximum when wild birds were compared against broilers. Several recent studies have reported QTLs associated with chicken growth traits in this region [14].

In total, eleven putative sweeps including *IGF1* had Z*F*_*ST*_ -scores more extreme than that of yellow skin/*BCO2* (Table 3), four of which were localized in regions lacking annotated genes. Other signals overlapped with *HNF4G*, *TBXAS1*, *GLP1R* and *GJD2*. A particularly interesting signal was found in the comparison of RJF/Commercials, and was localized at the distal end of GGA14 (Z*F*_*ST*_ = 9.65) coinciding a gene-rich region. This signal was further supported by analysis of the differences in nucleotide diversity between wild and domestic chicken (*ΔPi*) that revealed a high degree of fixation in domestic chicken in this window on GGA14 (see section ‘Analysis of fixation’ and Fig 2B), therefore we decided to further evaluate this signal.

A closer look at the GGA14 sweep (Fig 2C) revealed three separate signals emerging from the region. The window with the strongest signal (Z*F*_*ST*_ = 9.65) occurs in a window with a very high SNP density (nSNPs = 1,798) and overlaps the genes *JPT2* (Jupiter microtubule associated homolog 2) and *CRAMP1* (cramped chromatin regulator homolog 1). The signal reflects strong genetic differentiation between RJF and all domestic chickens (Fig 2). *JPT2* (also known as *HN1L)* shows high sequence conservation among vertebrates and are proposed to be involved in embryo development [15]. A study in *Drosophila melanogaster* showed that a *CRAMP1* homolog is involved in epigenetic regulation of gene expression [16].

The second signal (GGA14: 14.28–14.32 Mb, *ZF*_*ST*_ = 8.4) overlaps cyclin F (*CCNF*) and the third signal (GGA14:14.78–14.82, *ZF*_*ST*_ = 9.8) hits VPS35L (Vacuolar protein sorting-associated protein 35 like).

We explored these genes for function-altering mutations and identified 6 highly differentiated SNPs (ΔAF ≥ 0.7) between RJF and commercial populations, all residing in *CCNF*, annotated as missense mutations, (S10 Table), one of which was predicted to be deleterious.

#### b. Analysis of fixation

To extend the analysis of loci under selection during domestication, we compared the level of nucleotide diversity between wild birds and commercial lines. For this analysis, we included the six populations comprising sequence data from single individuals (see Table 1). We computed absolute values of the difference in nucleotide diversity (*ΔPi*) between groups of wild vs. commercial birds (RJFs vs. Coms) in every window and normalized the results by using a Z-score normalization (*ZΔPi* = (*ΔPi_win_*−*ΔPi_genome_*)/*σ*(*ΔPi_genome_*)). The most outstanding signal of Z|ΔPi| occurs on GGA14 overlapping the sweep signal encompassing the *JPT2* and *CRAMP1L* genes (Fig 2).

**Fig 3:**
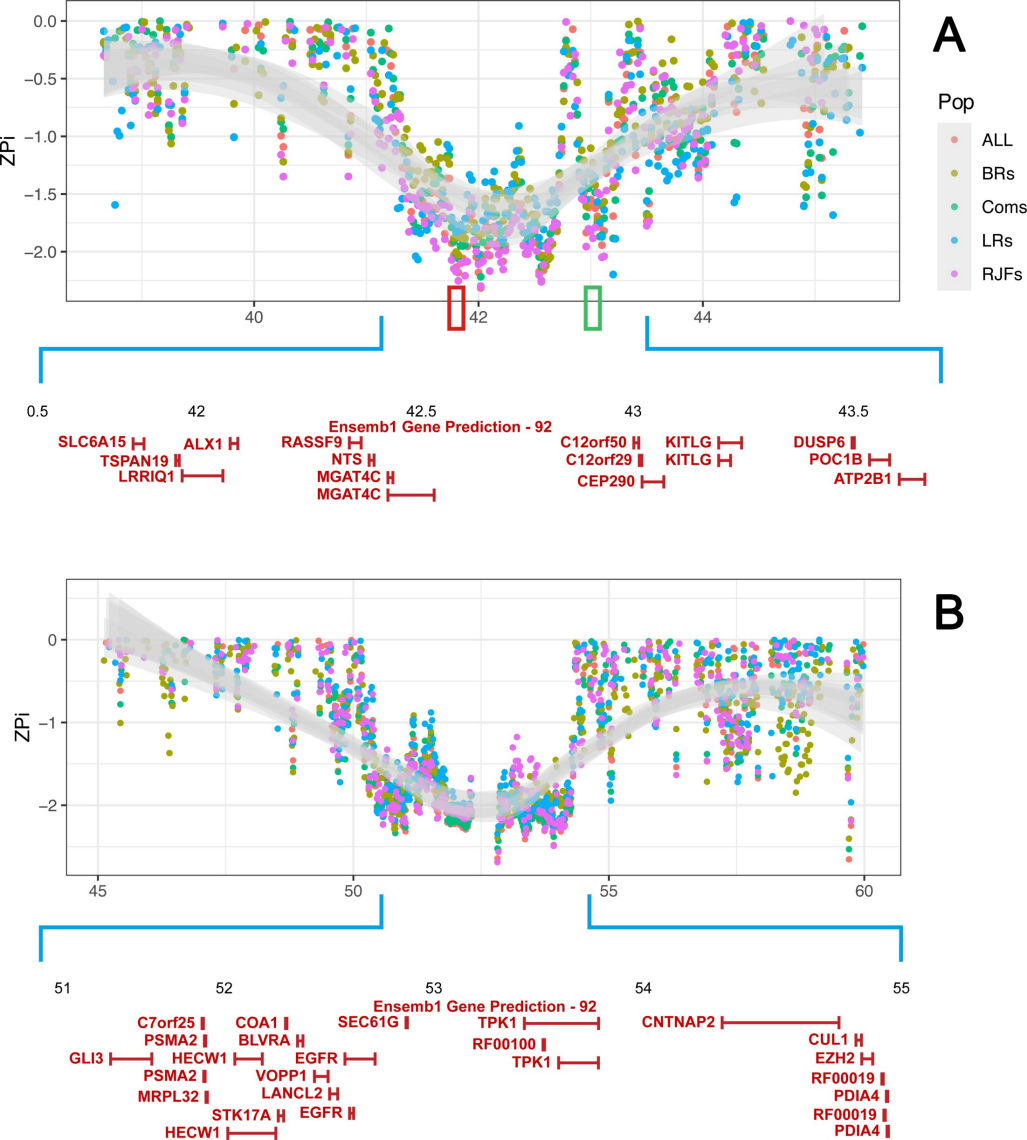
A graphical illustration of regions with extremely low nucleotide diversity across populations on GGA1 and GGA2. In panel A, two regions of high homozygosity are centered over *ALX1* and *KITLG* on GGA1. Red and green rectangles, respectively display the chromosomal positions of *ALX1* and *KITLG*. Panel B visualizes an extensive putative sweep on GGA2 overlapping the *EGFR* locus. Each dot represents a 40 kb window. The standard errors of ZPi-scores in each window across scans are smoothed over the region in grey. Nucleotide diversity was estimated for RJFs (two red jungle fowl populations), Coms (four commercial lines), BRs (the two commercial broiler lines, BRA and BRB), LRs (two layer populations, BL and WL) and ALL (all six populations of RJFs and commercials).

In a further step we estimated nucleotide diversity for groups of birds as well as all six populations of RJFs and commercials (S5A and S5B Fig). The latter scan may identify adaptive selection that happened prior to domestication in those cases where there is no significant genetic differentiation between populations but a reduction in nucleotide diversity in all of them. Density plots indicate no outlying signal at the negative tail of the diversity distributions implying the absence of aberrant local diversity across the genome, an observation that emerges from genomic distribution of diversity scores as well (see S5C Fig). At the local scale however, we noticed extensively swept regions that persisted across multiple consecutive windows and span over hundreds of kilobases (Fig 3). Two particularly interesting selective sweeps that are present in all populations overlap the genes for ALX Homeobox 1 *(ALX1)* and KIT Ligand *(KITLG)* on GGA1. The reason for classifying these as two separate sweeps is that they are separated by a highly variable region. The *ALX1* is responsible for beak shape variation among Darwin's finches [17]. The *KITLG* is a major determinant of pigmentation, which plays an important role in camouflage and sexual display [18]. As shown in Fig 3A, this is a fairly large region with an unusually low nucleotide diversity and we cannot rule out the possible involvement of other genes residing in the region contributing to the observed pattern. However, the two emerging valleys of homozygosity are evidently centred over *ALX1* and *KITLG*. The results suggest that beak morphology and pigmentation traits may have been under selection in chicken prior to domestication. Another noticeable sweep is located on GGA2 spanning over a ~3.5 Mb region harbouring 25 genes (Fig 3B).

### Genomic enrichment of functional variants

**Fig 4:**
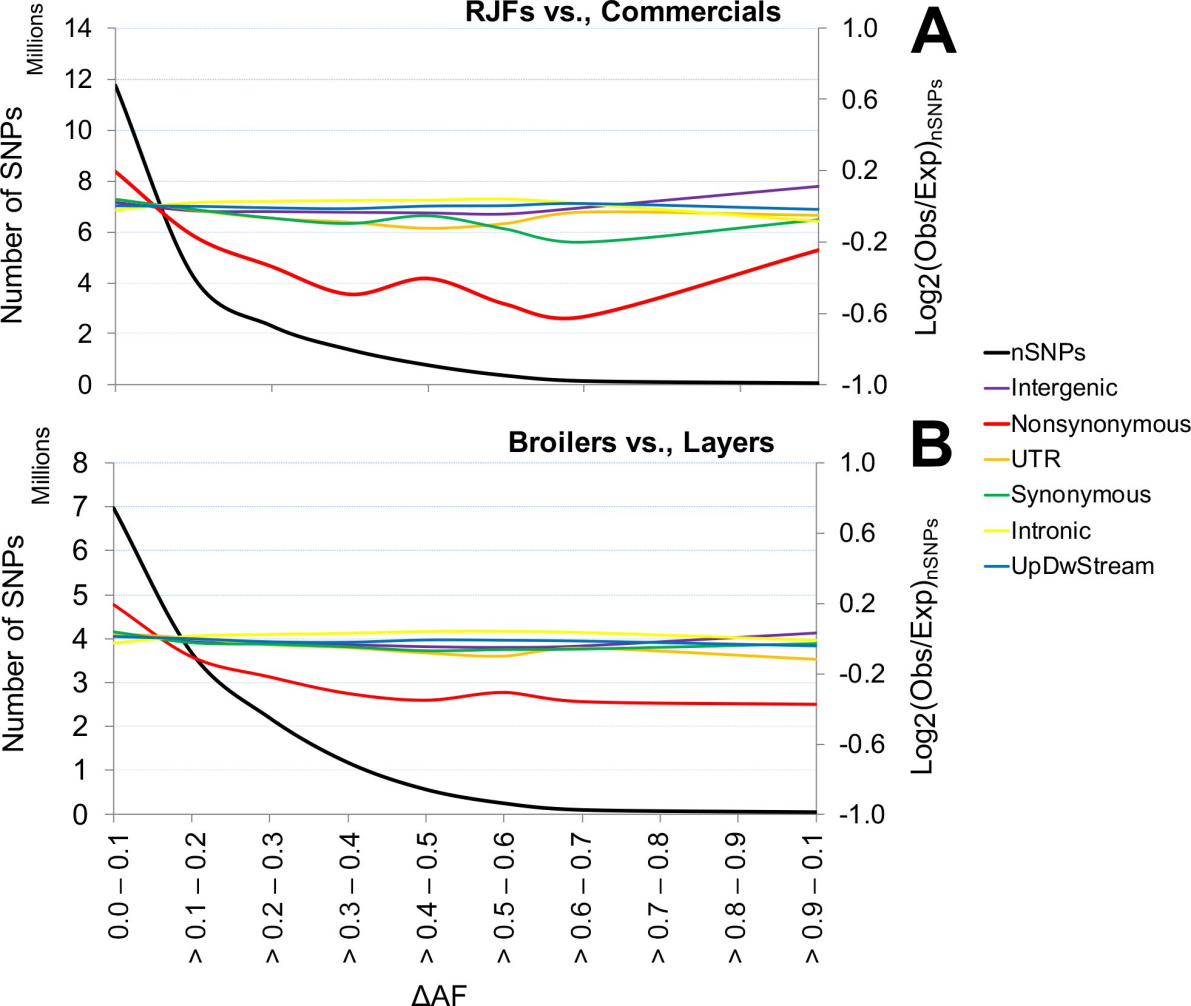
Analysis of enrichment for different categories of SNPs. Panel A and B represent the contrasts ‘RJFs vs. Coms’ and ‘BRs vs. LRs’, respectively. UpDwStream stands for sites residing 5 kb up- and downstream of genes. The black line represents the total number of SNPs in each ΔAF bin and colored lines represent log2 fold changes of the observed SNP count for each category in each bin against the expected SNP count.

The extensive SNP data combined with annotation information for each single site enabled us to explore the genomic distribution of sequence polymorphisms showing strong genetic differentiation between wild and domestic chicken as well as between broilers and layers. We carried out enrichment analyses to identify categories of SNPs showing differentiation between groups of birds. The absolute allele frequency difference (ΔAF) was calculated for different categories of SNPs in four contrasts (1) RJFs vs. Coms, (2) BRs vs. LRs, (3) RJFs vs. BRs and (4) RJFs vs. LRs and these ΔAF-values were sorted into 10 bins of allele frequency (ΔAF 0–0.1, etc.) to test for possible enrichment of variants in different annotation categories among SNPs showing strong differentiation. In all contrasts the great majority of SNPs showed a ΔAF<0.10 (Figs 4 and S6, S11–S14 Tables). This implies lack of differentiation between groups of birds at most loci, whereas a small percentage of variants, including those under selection showed highly significant differentiation.

The intensity of adaptive and purifying selection varies across the genome according to the functional properties; as such intergenic sequences evolve relatively more freely than protein-coding sequences. We observed a marked decline in relative abundance of missense substitutions showing a steady decrease above ΔAF = 0.2 in all contrasts (Fig 4). SNPs with marked allele frequency differences (ΔAF≥0.7) between wild and commercial chicken demonstrate a highly significant deficiency of missense mutations (P<2.5×10^−6^). We argue that this sharp decline in the proportion of differentiated missense substitutions represents purifying selection that reduces the frequency of slightly deleterious mutations affecting production and/or health. Thus, SNPs showing strong genetic differentiation between wild and domestic chickens are enriched for selectively neutral variants that have changed in frequency due to genetic drift as indicated by the enrichment of intergenic SNPs (P<0.0001) among variants with ΔAF>0.7.This result is in sharp contrast to recently reported data for the Atlantic herring where a similar analysis of high ΔAF SNPs showed a highly significant enrichment of missense mutations and other functionally important variants in a species with huge population size and a minimum amount of genetic drift [19].

The increase of log2 values for the contrast RJF vs. Coms and the flat curve for BRs vs. LRs (Fig 4) indicate most likely that a fraction of the missense mutations has been under positive selection during domestication. Therefore, we decided to focus on the highly differentiated missense variants (e.g., ΔAF>0.70), which were only 262 and 188 in the contrast ‘RJFs vs. Coms’ and ‘BRs vs. LRs’, respectively. All strongly differentiated missense variants in all four contrasts are compiled in S15–S18 Tables. Within the list of high ΔAF SNPs we observed multiple missense variants occurring in the same gene. For example, the 262 missense substitutions with ΔAF ≥ 0.70 in the RJFs vs. Coms contrast occur in only 189 different genes and the corresponding figure for the contrast BRs vs. LRs is 188 missense substitutions in 150 genes. This result may reflect hitchhiking or possibly the evolution of alleles composed of multiple causal variants affecting the function of the same gene as previously documented in domestic animals [20].

We used the hypergeometric test of FUNC [21] to perform a gene ontology enrichment analysis based on the list of all genes embedding differentiated missense mutations and found no significant overrepresentation of any particular biological process. Nevertheless, we noted that some of these variants occur in genes affecting domestication or production-related traits (Table 4). However, as most genes have pleiotropic effects, selection may possibly act on other functional effects of these genes than those highlighted here. In the following sections, we highlight some results from these analyses.

Evolution of pigmentation traits from wild to domestic type is one of the most striking changes during domestication [20]. Traits associated with visual appearance in domestic chicken have been artificially selected for aesthetic reasons and as a trademark in establishing distinct breeds. In the enrichment analysis of ‘RJFs vs. Coms’, two of the missense mutations with the highest ΔAF occur in the *CORIN* (AF_RJFs_ = 0.09 and AF_Coms_ = 0.96) and in *Ski2 Like RNA Helicase 2* (*SKIV2L2*, AF_RJFs_ = 0.71 and AF_Coms_ = 0.00) genes. *CORIN* is a modifier of Agouti signalling protein (ASIP) in dermal papilla and its absence causes ASIP activity being prolonged leading to lighter coat color in mice [22]. *SKIV2L2* regulates melanoblast proliferation during early stages of melanocyte regeneration [23]. Thus, both genes are involved in the pigmentation process. However, no genotype-phenotype association has yet been established for *CORIN* and *SKIV2L2* in chicken.

Among the top ΔAF alleles in the ‘RJFs vs. Coms’ contrast is the gene encoding sperm flagellar protein 2 (*SPEF2*, AF_RJFs_ = 0.03 and AF_Coms_ = 0.82). *SPEF2* is implicated in feather development. In contrast to the modern chicken, jungle fowl use feathers for flight and thermoregulation, both of which are more crucial in wild birds than in commercial chicken maintained in a controlled environment. However, thermoinsulation must have been an important trait in domestic chicken in the past when birds were kept in unheated stables in cold climate. Furthermore, *SPEF2* is a major candidate gene for chicken early- and late-feathering [24], which is an economically important trait in the poultry industry since it can be used to sex chickens, and likely another reason for the differentiation of this mutation through linked selection. Two other notable mutations in this contrast overlapped the GLI Family Zinc Finger 3 (*GLI3*, AF_RJFs_ = 0.03 and AF_Coms_ = 0.79) and the Kinesin Family Member 7 (*KIF7*, AF_RJFs_ = 0.03 and AF_Coms_ = 0.82) genes, both involved in Sonic hedgehog (Shh) signaling pathway that controls the normal shaping of many tissues and organs during embryogenesis including limb and wing development [25, 26]. Further genetic and functional studies of these allelic variants are necessary to verify their possible contribution to chicken domestication.

Coding SNPs with ΔAF≥0.7 in the contrast between BRs vs. LRs also included interesting candidate mutations. For example, a missense mutation of extreme ΔAF (AF_BRs_ = 0.14 and AF_LRs_ = 0.86), occur in the Leptin receptor gene (Table 4), whose function in regulating feed intake and body weight is well documented in mammals [27, 28] whereas the role of leptin-leptin receptor interaction for metabolic regulation in birds is not yet clear [29]. Another particularly interesting substitution in this contrast overlaps the multiple epidermal growth factor 10 gene (*MEGF10*, AF_BRs_ = 0.82 and AF_LRs_ = 0.00) on GGA8, known to function as a myogenic regulator of satellite cells in skeletal muscle [30]. Mutations in *MEGF10* have previously been shown to cause an unusual combination of dystrophic and myopathic features leading to the weak muscles in humans [30, 31], suggesting that the mutation reported here may affect muscle growth in broilers. The fact that different broiler lines have a high frequency of the variant allele at this locus is consistent with this suggestion. Other notable mutations in this contrast were found in the *IGSF10* gene implicated in age at puberty [32] and *PLEKHM1* with a suggested role in osteoporosis [33].

This paper reports the discovery and characterization of over 20 million SNPs from the chicken genome with the goal to delineate those with potential functional consequences—either having adaptive advantages or deleterious effects. To our knowledge, this is so far the largest study of its kind in chicken as a large number of individuals have been sequenced and a large number of sequence variants were detected. As many as 34% (n = 7,146,383) of the SNPs had not been reported before. The results revealed a subtle differentiation between wild and modern chicken at most loci, whereas a small percentage of loci showed strong differentiation. Analysis of selection provided a comprehensive list of several tens of independent loci that are likely to have contributed to domestication or improving production. We confirmed strong differentiation between red jungle fowl and domestic chickens at the previously reported *BCO2* and *TSHR* loci. We identified 34 putative selective sweeps co-localized with, among others, *KITLG*, *ALX1*, *IGF1*, *DLK1*, *JPT2* and *CRAMP1*. Single SNP contrasts between groups of birds revealed several highly differentiated coding variants, in genes such as *CORIN* and *SKIV2L2* involved in pigmentation and *LEPR*, *MEGF10* and *SPEF2* possibly affecting traits relevant for animal production. SNPs with marked allele frequency differences between wild and domestic chicken showed a highly significant deficiency of the proportion of missense mutations (P<2.5×10^−6^).

## Methods

### Ethics statement

Samples were either taken from a DNA bank established at Friedrich-Loeffler-Institut during the EC project AVIANDIV (1998–2000; EC Contract No. BIO4-CT98-0342, https://aviandiv.fli.de) or as part of the SYNBREED project (2009–2014, Funding ID: 0315526; http://www.synbreed.tum.de/) where sampling was done in strict accordance to the German Animal Welfare regulations (33.9-42502-05-10A064) and with written consent of the animal owners.

### Genetic material

Three groups of birds were included in the study (1) red jungle fowls (*Gallus gallus gallus*, RJFs), (2) broilers (BRs) and (3) layers (LRs) (Table 1). The RJFs were sampled from two geographical regions, Thailand (RJFt) and India (RJFi). The RJFt consisted of 25 DNA samples collected within a European collaborative research project AVIANDIV (https://aviandiv.fli.de/). RJFt was randomly down-sampled from ~150 RJFs caught in northern Thailand in 1997 and maintained since with random mating over four flocks; given the place and date, the RJFt samples likely have seen some contamination from domestic or feral populations prior to collection [34]. The DNA samples from RJFt were collected in 1999. For further information on the behaviour and morphology of these birds we refer to the AVIANDIV project webpage. The RJFi population involved 10 individuals of the Richardson line, originating from RJF caught in India in the 1960´s. This population has been extensively studied [35–37], and appears to have been established from a wild population prior to major genetic contamination of red jungle fowl populations, such that it may represent a unique RJF line that is at least largely free of influence from domestic stocks. The second and third group of birds represent commercial chicken, comprising three broiler and three layer populations, respectively. The broilers (BRs) were represented by 20 DNA samples of each of two lines (BRA and BRB) established independently and previously collected as part of the AVIANDIV project. BRA was a sire line belonging to the company Indian River International (Texas) established in 1980 and closed since with a breeding population size of >10,000 birds. BRB was another sire line originally from France, developed in 1970 with a breeding population size varying between 10,000 to 70,000. The broiler group further involved a pooled sample of 25 birds from AVIANDIV’s broiler sire line D, hereafter denoted BRpD. This is a sire line originally from UK, established in 1974 and closed since with unknown population size. In the layer group (LRs), data from 25 birds each from purebred white (WL) and brown (BL) egg laying populations, sequenced in the frame of the SYNBREED project (http://www.synbreed.tum.de/index.php?id=2), were included. WL and BL birds represent parental lines of the LOHMANN Tierzucht GmbH that are originally established from White Leghorn and Rhode Island Red, respectively. Moreover, we used pooled sequence data of 48 birds from Rhode Island White (RWp), a crossbred layer population collected by the AVIANDIV project.

### DNA sequencing, alignment and variant calling

Sequencing libraries of 300–500 bp fragments were constructed for each individual sample using Illumina Nextera Library preparation kits. Sequencing of RJFt, BRA and BRB was conducted using an Illumina HiSeq 2500 machine and 2x126 bp paired-end reads were generated. RJFi, WL and BL along with the three DNA pools (RWp, BRpB and BRpD) were sequenced with 2x101 bp paired-end reads (see Table 1). All reads were mapped against the reference genome assembly Galgal5 [38] using the Burrows-Wheeler aligner (bwa-0.7.12) [39]. Duplicate reads were masked during pre-processing using the Picard tool set (version 2.0.1).

We identified SNPs following the recommendations of best practices workflow for variant discovery analysis using GATK [40]. Briefly, after recalibrating for base quality scores, BAM files were fed into the GATK-HaplotypeCaller tool which is capable of calling SNPs and INDELs simultaneously via local *de-novo* assembly of haplotypes in a region. After generating 127 GVCF files for individual and pooled samples, they were called simultaneously using the GenotypeGVCFs module. Raw vcf files were then filtered and used for downstream analyses. S1 Fig presents a summary of SNPs called based on different sequencing parameters.

### Data preparation

The number of detected variants was 26,290,203 which included 3,442,027 INDELs and 1,024,944 multi-allelic sites. Raw vcf files from both individuals and pools were filtered primarily based on the following parameters. Variants were removed with QualByDepth (QD) < 4.0, 300 > depth > 2200, Quality < 30, mapping quality (MQ) < 40.0, MQRankSum < -10, ReadPosRankSum < -7.0, Fisher Strand > 60.0, ReadPosRankSum > 7, BaseQRankSum < -6, BaseQRankSum > 6". Cluster Size and ClusterWindowSize were set to 4 and 10, respectively. For the subsequent analyses we used only bi-allelic SNPs on autosomes and chromosomes W and Z. In total, 21,190,795 SNPs were retained for downstream analysis.

### Analysis of population structure and relatedness

The R packages SNPRelate and gdsfmt [41] were used for principal component analysis of relatedness using identity-by-descent measures estimated from all SNPs.

### Annotation of genetic variants

SnpEff (v.3.4) [42] was used to annotate variants according to their functional categorization which included the following categories 5 kb up- and down-stream of a gene, intergenic, missense, synonymous, intronic, 3' untranslated regions, 5' untranslated regions, stop gain and stop loss. Variants in the up- and down-stream regions and in the 3' UTR, 5' UTR regions were merged into the single categories.

The online tool Ensemble Variant Effect Predictor (VEP, webpage: http://www.ensembl.org/info/docs/tools/vep/index.html)) [43], was used to predict SIFT-scores for amino-acid altering substitutions.

### Enrichment analysis

The enrichment analysis was conducted as previously described in [44] for four contrasts (1) RJFs vs. commercial and (2) BRs vs. LRs, (3) RJFs vs. BRs and (4) RJFs vs. LRs. First we estimated the allele frequency (AF) of each SNP based on the proportion of high-quality reads supporting the non-reference allele. To ensure an unbiased estimation of AF several filters were employed to remove low quality SNPs and uncertain genotypes. In individually sequenced populations, loci with genotype quality < 20 were set to no.call and allele frequencies were estimated only for sites with >50% of the individuals genotyped. Because of low coverage, we treated the population RJFi as a pool in this analysis. In all pools SNPs with allelic depth <50% of mean coverage were set to no.call. Then, for each contrast, allele frequencies of intra-group populations were averaged and used to estimate the absolute value of allele frequency difference (ΔAF) for every single variant. The SNPs were then sorted into different bins of ΔAF (e.g., 0–0.1, >0.1–0.2, etc.) representing the allele frequency difference between populations. The expected number of SNPs for each category in each bin was calculated as p(category) X n(bin), where p(category) is the proportion of a specific SNP category in the entire genome and n(bin) is the total number of SNPs in a given bin. Finally, log2 fold changes of the observed SNP count for each category in each bin were compared against the expected SNP count and statistical significance of the deviations from the expected values was tested with a standard χ^2^ test.

### Detecting selective sweeps

Evidence of positive selection was investigated in two steps. First, we explored differentiation of loci between the following combinations of populations. (1) RJFs vs. Commercials, (2) BRs vs. LRs, (3) RJFs vs. BRs and (4) RJFs vs. LRs. We estimated *F*_*ST*_ [45] for each of these four contrasts. To reduce locus-to-locus variation in the inference of selection we averaged single SNP values for sliding windows of 40 kb with 20 kb overlap across chicken chromosomes. Window-based *F*_*ST*_ values were then normalized and windows in the outlier tail Z*F*_*ST*_ > 6 were identified as selection candidates for domestication and genetic improvement in commercial populations.

In the second step, we searched the genome for regions with high degrees of fixation. To this purpose, the nucleotide diversity (*Pi*) was compared between RJF and commercial birds as a signature of selection during domestication. Different window sizes were tested but did not change the consistent picture of the signals. A window size of 40 kb was selected in accordance to the differentiation analysis. The *Pi* values were then normalized. Analysis of fixation involved six populations for which individually sequenced data were available. As such, nucleotide diversity was estimated for RJFs (two red jungle fowl populations), commercials (four commercial lines), broilers (the two commercial broiler lines, BRA and BRB), LRs (two layer populations, BL and WL) and ALL (all six populations of RJFs and commercials).

Gene ontology enrichment analyses, contrasting differentiated genes against a genomic background gene set, were performed using the hypergeometric test of FUNC [21].

## Supporting information

S1 Table The frequency distribution of layer-specific SNPs (segregating only in white layer, brown layer and Rhode Island White) in different annotation categories. (DOCX)

S2 Table List of layer-specific missense variants and corresponding genes (mean AF*>0.5). (DOCX)

S3 Table The frequency distribution of broiler-specific SNPs (segregating only in BRA, BRB and BRpD) in different annotation categories. (DOCX)

S4 Table List of broiler-specific missense SNPs and corresponding genes (mean AF*>0.5). (DOCX)

S5 Table Genome-wide *F_ST_*-scores averaged over 40 kb windows between wild and commercial birds (RJFs vs. Coms). (XLS)

S6 Table Genome-wide *F_ST_*-scores averaged over 40 kb windows between broilers and layers (BRs vs. LRs). (XLS)

S7 Table Genome-wide *F_ST_*-scores averaged over 40 kb windows between wild birds and broilers (RJFs vs. BRs). (XLS)

S8 Table Genome-wide *F_ST_*-scores averaged over 40 kb windows between wild birds and layers (RJFs vs. LRs). (XLS)

S9 Table The list of windows exceeding *ZF_ST_* ≥ 4 in either differentiation comparison. (XLS)

S10 Table List of missense substitution revealed in the top putative selective sweep on GGA14. (DOCX)

S11 Table Distribution of SNPs with functional annotation in different delta allele frequency bins between two wild and six commercial populations (‘RJFs vs. Commercials’). (DOCX)

S12 Table Distribution of SNPs with functional annotation in the delta allele frequency bins between three broiler and three layer populations (BRs vs. LRs). (DOCX)

S13 Table Distribution of SNPs with functional annotation in different delta allele frequency bins between two wild and three layer populations (RJFs vs. LRs). (DOCX)

S14 Table Distribution of SNPs with functional annotation in different delta allele frequency bins between two wild and four broiler populations (RJFs vs. BRs). (DOCX)

S15 Table Differentiated missense variants (ΔAF>0.70) in the contrast RJF vs. commercials. (XLSX)

S16 Table Differentiated missense variants (ΔAF>0.70) in the contrast broilers vs. layers. (XLSX)

S17 Table Differentiated missense variants (ΔAF>0.70) in the contrast RJF vs. broilers. (XLSX)

S18 Table Differentiated missense variants (ΔAF>0.70) in the contrast RJF vs. layers. (XLSX)

S1 Fig Summary statistics of detected polymorphism. SNPs called according to the best practices workflow using GATK (McKenna et al., 2010). The quality parameters shown from left to right and top to bottom are: Phred Quality Score; Allele Frequency; Depth of Coverage; Base Quality Rank Sum; Clipping Rank Sum; Excess of Heterozygosity; Fisher Strand; Inbreeding Coefficient; Maximum Likelihood Expectation for the Allele counts; Maximum Likelihood Expectation for the Allele Frequency; Mapping Quality; Mapping Quality Rank Sum; Quality by Depth; Z-score from Wilcoxon rank sum test of Alt vs. Ref read position bias; Strand Odds Ratio and Allelic Number in called genotypes.
(TIF)

S2 Fig Allele frequency spectrum of SNPs with different annotation categories. Colored lines depict the distribution of alternative allele for SNPs in different annotation categories.
(TIF)

S3 Fig Clustering individuals based on genetic similarity. (TIF)

S4 Fig Analysis of genetic differentiation. (A) *F_ST_* among chromosomes in different contrasts of differentiation. The average *F_ST_* values varied both among chromosomes and between autosomes and chromosome Z. (B) Average *F_ST_* for different categories of SNPs. Function-altering variants such as stop-gain or loss as well as missense mutations show lower degrees of differentiation than other annotation categories. (C) Distribution of *ZF_ST_*-scores averaged over 40 kb windows in different contrasts.
(TIF)

S5 Fig Analysis of fixation. Panel A and B, respectively displays distribution of number of variants and ZPi scores estimated in 40 kb windows in steps of 20 kb in different groups of birds. Panel C provides a schematic representation of the genome-wide nucleotide diversity (ZPi-scores). Nucleotide diversity are estimated only for the six individually sequenced populations. Each dot represents a ZPi-score for a 40 kb window.
(TIF)

S6 Fig Analysis of enrichment for SNPs in different annotation categories in relation to delta allele frequencies (ΔAF). Panel A and B represent the contrasts ‘RJFs vs. LRs’ and ‘RJFs vs. BRs’, respectively. The Y axis represents number of SNPs. The black line represents the total number of SNPs in each ΔAF bin and the colored lines represent log2-fold changes of the observed SNP count for each category in each bin against the expected SNP count. UpDwStream stands for SNPs residing 5 kb up or downstream of genes.
(TIF)
