# Supplementary material for: Genetics of adaptation in modern chicken
Source: PLoS Genet. 2019 Apr 29;15(4):e1007989. doi: 10.1371/journal.pgen.1007989 (PMC6508745; doi:10.1371/journal.pgen.1007989)
Supplement: S1 Table — (DOCX) [file pgen.1007989.s001.docx]

| **Table S1. The frequency distribution of layer-specific SNPs (segregating only in white layer, brown layer and Rhode Island White)** **in different annotation categories.** | | | | | | | | | |
| --- | --- | --- | --- | --- | --- | --- | --- | --- | --- |
| Bin* | BinCount | UpDw | UTR | Intergenic | Missense | Syn | Intronic | StopG | StopL |
| 0-0.1 | 905 | 224 | 23 | 513 | 20 | 14 | 330 | 0 | 0 |
| 0.1-0.2 | 972 | 227 | 28 | 399 | 8 | 7 | 532 | 0 | 0 |
| 0.2-0.3 | 1002 | 175 | 14 | 410 | 6 | 5 | 557 | 0 | 0 |
| 0.3-0.4 | 1025 | 199 | 34 | 401 | 5 | 10 | 571 | 0 | 0 |
| 0.4-0.5 | 773 | 152 | 22 | 326 | 9 | 10 | 406 | 0 | 0 |
| 0.5-0.6 | 448 | 95 | 13 | 158 | 6 | 6 | 258 | 0 | 0 |
| 0.6-0.7 | 199 | 40 | 15 | 73 | 2 | 1 | 112 | 0 | 0 |
| 0.7-0.8 | 64 | 11 | 2 | 27 | 0 | 0 | 35 | 0 | 0 |
| 0.8-0.9 | 16 | 9 | 0 | 11 | 0 | 0 | 5 | 0 | 0 |
| 0.9-1 | 5 | 1 | 1 | 1 | 0 | 1 | 1 | 0 | 0 |
| Sum | 5409 | 1133 | 152 | 2319 | 56 | 54 | 2807 | 0 | 0 |
| *Bins of average allele frequency estimated across three layer populations for 5409 layers-specific variants. | | | | | | | | | |
