# Supplementary material for: Genetics of adaptation in modern chicken
Source: PLoS Genet. 2019 Apr 29;15(4):e1007989. doi: 10.1371/journal.pgen.1007989 (PMC6508745; doi:10.1371/journal.pgen.1007989)
Supplement: S2 Table — (DOCX) [file pgen.1007989.s002.docx]

**Table S2. List of layers-specific missense variants and corresponding genes (mean AF*>0.5).**

| **ID** | **Ref** | **Alt** | **BRA** | **BRB** | **BRpD** | **AF*** | **AA** | **SIFT** | **Gene** |
| --- | --- | --- | --- | --- | --- | --- | --- | --- | --- |
| 1_85029720 | T | C | 0.960 | 0.480 | 0.097 | 0.512 |  |  | ABI3BP |
| 1_195901648 | C | T | 0.100 | 0.957 | NA | 0.528 |  |  | CHRDL2 |
| 1_195901649 | A | G | 0.100 | 0.957 | NA | 0.528 |  |  | CHRDL2 |
| 18_6193836 | C | T | 0.460 | 0.420 | 0.853 | 0.578 |  |  | ANKFN1 |
| 19_4143485 | G | A | 0.783 | 0.500 | NA | 0.642 |  |  | RASA4 |
| 28_4782299 | C | T | 0.478 | 1.000 | 0.375 | 0.618 |  |  | KDM4B |
| 28_4952320 | G | A | 0.480 | 1.000 | 0.314 | 0.598 |  |  | C28H19ORF10 |
| 3_45726698 | C | A | 0.120 | 0.560 | 0.846 | 0.509 |  |  | MRPL18 |

* Average frequency of layer-specific missense variants across three layer populations
