# Supplementary material for: Genetics of adaptation in modern chicken
Source: PLoS Genet. 2019 Apr 29;15(4):e1007989. doi: 10.1371/journal.pgen.1007989 (PMC6508745; doi:10.1371/journal.pgen.1007989)
Supplement: S3 Table — (DOCX) [file pgen.1007989.s003.docx]

| **Table S3. The frequency distribution of broiler-specific SNPs (segregating only in BRA, BRB and BRpD) in different annotation categories.** | | | | | | | | | |
| --- | --- | --- | --- | --- | --- | --- | --- | --- | --- |
| Bin* | BinCount | UpDw | UTR | Intergenic | Missense | Syn | Intronic | StopG | StopL |
| 0-0.1 | 33700 | 7092 | 921 | 13137 | 216 | 287 | 19063 | 4 | 0 |
| 0.1-0.2 | 84025 | 17253 | 2557 | 32711 | 538 | 795 | 47228 | 1 | 1 |
| 0.2-0.3 | 52651 | 10590 | 1542 | 19852 | 342 | 543 | 30210 | 1 | 1 |
| 0.3-0.4 | 23175 | 4291 | 651 | 8903 | 135 | 211 | 13204 | 3 | 0 |
| 0.4-0.5 | 9156 | 1574 | 292 | 3466 | 46 | 84 | 5268 | 0 | 0 |
| 0.5-0.6 | 2763 | 522 | 93 | 1068 | 11 | 22 | 1570 | 1 | 0 |
| 0.6-0.7 | 1128 | 192 | 35 | 395 | 11 | 9 | 663 | 1 | 0 |
| 0.7-0.8 | 473 | 71 | 15 | 221 | 0 | 3 | 231 | 0 | 0 |
| 0.8-0.9 | 44 | 7 | 1 | 17 | 0 | 0 | 26 | 0 | 0 |
| 0.9-1 | 7 | 2 | 0 | 3 | 0 | 0 | 4 | 0 | 0 |
| Sum | 207122 | 41594 | 6107 | 79773 | 1299 | 1954 | 117467 | 11 | 2 |
| *Bins of average allele frequency estimated across three broiler populations for 207,122 broilers-specific variants. | | | | | | | | | |
