# Supplementary material for: Genetics of adaptation in modern chicken
Source: PLoS Genet. 2019 Apr 29;15(4):e1007989. doi: 10.1371/journal.pgen.1007989 (PMC6508745; doi:10.1371/journal.pgen.1007989)
Supplement: S4 Table — (DOCX) [file pgen.1007989.s004.docx]

**Table S4. List of broiler-specific missense SNPs and corresponding genes (mean AF*>0.5)**

| **ID** | **Ref** | **Alt** | **BRA** | **BRB** | **BRpD** | **AF*** | **AA** | **SIFT** | **Gene** |
| --- | --- | --- | --- | --- | --- | --- | --- | --- | --- |
| 1_180671124 | A | T | 0.500 | 0.275 | 0.761 | 0.512 |  |  | CWF19L2 |
| 18_590273 | G | A | 0.425 | 0.975 | 0.659 | 0.686 |  |  | MYH1E |
| 18_8020330 | G | A | 0.375 | 0.575 | 0.914 | 0.621 |  |  | ABCA9 |
| 2_26699857 | T | C | 0.475 | 0.800 | 0.412 | 0.562 |  |  | VWDE |
| 2_26703136 | C | T | 0.475 | 0.800 | 0.486 | 0.587 |  |  | VWDE |
| 2_26723519 | G | C | 0.475 | 0.800 | 0.432 | 0.569 |  |  | VWDE |
| 2_80236250 | A | G | 0.475 | 0.850 | 0.459 | 0.595 |  |  | ABCA13 |
| 22_2796439 | G | A | 0.625 | 0.868 | 0.529 | 0.674 |  |  | KANSL3 |
| 28_2777873 | C | T | 0.600 | 0.600 | 0.368 | 0.523 |  |  | LOC101751474 |
| 28_2794490 | G | T | 0.625 | 0.526 | 0.436 | 0.529 |  |  | MISP |
| 28_4088542 | C | T | 0.625 | 0.700 | 0.643 | 0.656 |  |  | ARHGEF18 |
| 3_17590421 | G | A | 0.400 | 0.800 | 0.600 | 0.600 |  |  | NVL |
| 5_5093245 | A | G | 0.550 | 0.600 | 0.706 | 0.619 |  |  | ELP4 |
| 5_10624038 | G | A | 0.250 | 0.700 | 0.829 | 0.593 |  |  | INSC |
| 5_12053911 | C | T | 0.475 | 0.725 | 0.620 | 0.607 |  |  | OTOG |
| 5_51849534 | A | C | 0.525 | 0.400 | 0.649 | 0.525 |  |  | CEP170B |
| 6_9401664 | G | A | 0.450 | 0.900 | 0.644 | 0.665 |  |  | ZNF511 |
| 7_36499783 | T | C | 0.375 | 0.700 | 0.462 | 0.512 |  |  | UPP2 |
| 8_6258405 | C | T | 0.750 | 0.475 | 0.655 | 0.627 |  |  | TOR3A |
| 8_7070578 | G | A | 0.625 | 0.675 | 0.614 | 0.638 |  |  | TNR |
| 9_16101482 | G | A | 0.625 | 0.900 | 0.489 | 0.671 |  |  | LOC107054090 |
| 9_16101488 | A | C | 0.625 | 0.900 | 0.543 | 0.689 |  |  | LOC107054090 |

*Average frequency of broiler-specific missense variants across three broiler populations.
