# Supplementary material for: Genetics of adaptation in modern chicken
Source: PLoS Genet. 2019 Apr 29;15(4):e1007989. doi: 10.1371/journal.pgen.1007989 (PMC6508745; doi:10.1371/journal.pgen.1007989)
Supplement: S10 Table — (DOCX) [file pgen.1007989.s010.docx]

| **Table S10. List of missense substitution revealed in the top putative sweep on GGA14.** | | | | | | | | | | | | | | | | | |
| --- | --- | --- | --- | --- | --- | --- | --- | --- | --- | --- | --- | --- | --- | --- | --- | --- | --- |
| **Pos** | **Ref** | **Alt** | **BRA** | **BRB** | **RJFt** | **BL** | **WL** | **RJFi** | **RWp** | **BRpD** | **ΔRJFsComs** | **ΔRJFsBRs** | **ΔRJFsLRs** | **ΔBRsLRs** | **AA^a^** | **Gene** | **SIFT** |
| 14296774 | A | G | 0.15 | 0.00 | 0.84 | 0.00 | 0.00 | 1.00 | 0.93 | 0.00 | 0.74 | 0.87 | 0.61 | 0.26 | I/M | CCNF | Deleterious (0.01) |
| 14296806 | A | C | 0.11 | 0.00 | NA | 0.00 | 0.00 | 1.00 | 0.92 | 0.00 | 0.83 | 0.97 | 0.70 | 0.27 | Y/S | CCNF | Tolerated (0.36) |
| 14297351 | A | C | 0.08 | 0.00 | NA | 0.00 | 0.00 | 1.00 | 0.00 | 0.68 | 0.87 | 0.75 | 1.00 | 0.25 | T/P | CCNF | tolerated(0.35) |
| 14299501 | A | G | 0.11 | 0.00 | 0.68 | 0.00 | 0.00 | 0.89 | 0.00 | 0.36 | 0.71 | 0.63 | 0.78 | 0.16 | T/A | CCNF | Tolerated_low_confidence (0.95) |
| 14299502 | C | T | 0.11 | 0.00 | 0.68 | 0.00 | 0.00 | 0.89 | 0.00 | 0.36 | 0.71 | 0.63 | 0.78 | 0.15 | T/I | CCNF | Tolerated_low_confidence (0.4) |
| 14299516 | C | G | 0.11 | 0.00 | 0.68 | 0.00 | 0.00 | 0.89 | 0.00 | 0.34 | 0.71 | 0.64 | 0.78 | 0.15 | P/A | CCNF | Tolerated_low_confidence(0.15) |
| Summary statistics presented for RJFt=red jungle fowl (Thailand), RJFi=red jungle fowl (India), BL=Brown layer, WL=White layer, RWp, Rhode and White pool, BRA=Broiler line A, BRB=Broiler line B and BRpD= Broiler line pool D.  ^a^Shift in amino acid | | | | | | | | | | | | | | | | | |
