# Supplementary material for: Genetics of adaptation in modern chicken
Source: PLoS Genet. 2019 Apr 29;15(4):e1007989. doi: 10.1371/journal.pgen.1007989 (PMC6508745; doi:10.1371/journal.pgen.1007989)
Supplement: S11 Table — (DOCX) [file pgen.1007989.s011.docx]

**Table S11. Distribution of SNPs with functional annotation in different delta allele frequency bins between two wild and six commercial populations (‘RJFs vs. Commercials’).**

| **Bin** | **BinCount** | **UpDw** | **UTR** | **Intergenic** | **Missense** | **Syn** | **Intronic** | **StopG** | **StopL** |
| --- | --- | --- | --- | --- | --- | --- | --- | --- | --- |
| 0-0.1 | 11743292 | 2430944 | 335849 | 4810911 | 72276 | 121742 | 6383962 | 641 | 104 |
| 0.1-0.2 | 4227562 | 873408 | 115918 | 1676607 | 20275 | 42157 | 2363726 | 161 | 26 |
| 0.2-0.3 | 2304944 | 473405 | 61155 | 911325 | 9810 | 22222 | 1295400 | 57 | 10 |
| 0.3-0.4 | 1369344 | 280426 | 35782 | 539908 | 5232 | 12926 | 772168 | 33 | 7 |
| 0.4-0.5 | 763605 | 157899 | 19496 | 300358 | 3103 | 7428 | 431152 | 22 | 4 |
| 0.5-0.6 | 352977 | 73121 | 9181 | 138267 | 1297 | 3260 | 200203 | 8 | 0 |
| 0.6-0.7 | 140461 | 29333 | 3819 | 56417 | 492 | 1233 | 78192 | 5 | 2 |
| 0.7-0.8 | 44566 | 9133 | 1220 | 18868 | 175 | 417 | 23756 | 2 | 0 |
| 0.8-0.9 | 10039 | 1937 | 242 | 4571 | 39 | 77 | 5074 | 0 | 0 |
| 0.9-1 | 3132 | 716 | 88 | 1745 | 48 | 59 | 1204 | 0 | 0 |
| Sum | 20959922 | 4330322 | 582750 | 8458977 | 112747 | 211521 | 11554837 | 929 | 153 |
