# Supplementary material for: Genetics of adaptation in modern chicken
Source: PLoS Genet. 2019 Apr 29;15(4):e1007989. doi: 10.1371/journal.pgen.1007989 (PMC6508745; doi:10.1371/journal.pgen.1007989)
Supplement: S12 Table — (DOCX) [file pgen.1007989.s012.docx]

**Table S12. Distribution of SNPs with functional annotation in the delta allele frequency bins between three broiler and three layer populations (BRs vs. LRs).**

| **Bin** | **BinCount** | **UpDw** | **UTR** | **Intergenic** | **Missense** | **Syn** | **Intronic** | **StopG** | **StopL** |
| --- | --- | --- | --- | --- | --- | --- | --- | --- | --- |
| 0-0.1 | 6967584 | 1473616 | 196057 | 2848985 | 40635 | 71984 | 3797282 | 380 | 56 |
| 0.1-0.2 | 3672179 | 770135 | 101385 | 1453124 | 17438 | 36353 | 2056434 | 131 | 27 |
| 0.2-0.3 | 2189712 | 453810 | 58932 | 860666 | 9605 | 21542 | 1234312 | 52 | 12 |
| 0.3-0.4 | 1176819 | 243444 | 31308 | 460286 | 4837 | 11461 | 666436 | 30 | 5 |
| 0.4-0.5 | 563786 | 117668 | 14688 | 218769 | 2256 | 5406 | 321565 | 15 | 3 |
| 0.5-0.6 | 248654 | 51837 | 6398 | 96226 | 1026 | 2396 | 141896 | 8 | 0 |
| 0.6-0.7 | 98515 | 20472 | 2615 | 38341 | 392 | 951 | 55959 | 1 | 0 |
| 0.7-0.8 | 34205 | 6834 | 886 | 13408 | 126 | 330 | 19424 | 0 | 0 |
| 0.8-0.9 | 9134 | 1886 | 224 | 3839 | 29 | 82 | 4970 | 0 | 0 |
| 0.9-1 | 4411 | 1016 | 103 | 2310 | 33 | 59 | 1915 | 0 | 0 |
| Sum | 14964999 | 3140718 | 412596 | 5995954 | 76377 | 150564 | 8300193 | 617 | 103 |
