# Supplementary material for: Genetics of adaptation in modern chicken
Source: PLoS Genet. 2019 Apr 29;15(4):e1007989. doi: 10.1371/journal.pgen.1007989 (PMC6508745; doi:10.1371/journal.pgen.1007989)
Supplement: S13 Table — (DOCX) [file pgen.1007989.s013.docx]

**Table S13. Distribution of SNPs with functional annotation in different delta allele frequency bins between two wild and three layer populations (RJFs vs. LRs).**

| Bin | **BinCount** | **UpDw** | **UTR** | **Intergenic** | **Missense** | **Syn** | **Intronic** | **StopG** | **StopL** |
| --- | --- | --- | --- | --- | --- | --- | --- | --- | --- |
| 0-0.1 | 10043701 | 2061465 | 282688 | 4110529 | 58870 | 102926 | 5471022 | 500 | 80 |
| 0.1-0.2 | 3894223 | 803526 | 106945 | 1547413 | 18733 | 38737 | 2174476 | 152 | 24 |
| 0.2-0.3 | 2405208 | 492656 | 64102 | 950229 | 10440 | 23336 | 1351641 | 67 | 10 |
| 0.3-0.4 | 1537731 | 315692 | 40310 | 607246 | 6231 | 14624 | 865598 | 49 | 14 |
| 0.4-0.5 | 910008 | 190620 | 24238 | 357312 | 3926 | 8950 | 513740 | 21 | 2 |
| 0.5-0.6 | 490336 | 102227 | 12868 | 192229 | 1764 | 4574 | 277678 | 9 | 4 |
| 0.6-0.7 | 235871 | 49811 | 6401 | 92462 | 840 | 2250 | 133389 | 8 | 0 |
| 0.7-0.8 | 98336 | 20924 | 2649 | 38541 | 398 | 938 | 55620 | 2 | 1 |
| 0.8-0.9 | 34704 | 7367 | 966 | 14325 | 139 | 326 | 18919 | 2 | 0 |
| 0.9-1 | 11096 | 2425 | 277 | 5237 | 85 | 144 | 5339 | 0 | 0 |
| Sum | 19661214 | 4046713 | 541444 | 7915523 | 101426 | 196805 | 10867422 | 810 | 135 |
