# Supplementary material for: Genetics of adaptation in modern chicken
Source: PLoS Genet. 2019 Apr 29;15(4):e1007989. doi: 10.1371/journal.pgen.1007989 (PMC6508745; doi:10.1371/journal.pgen.1007989)
Supplement: S14 Table — (DOCX) [file pgen.1007989.s014.docx]

**Table S14. Distribution of SNPs with functional annotation in different delta allele frequency bins between two wild and four broiler populations (RJFs vs. BRs).**

| Bin | **BinCount** | **UpDw** | **UTR** | **Intergenic** | **Missense** | **Syn** | **Intronic** | **StopG** | **StopL** |
| --- | --- | --- | --- | --- | --- | --- | --- | --- | --- |
| 0-0.1 | 10969794 | 2269255 | 314203 | 4468606 | 65472 | 112751 | 5991360 | 572 | 94 |
| 0.1-0.2 | 3926601 | 816344 | 107546 | 1556723 | 19149 | 39482 | 2195815 | 156 | 22 |
| 0.2-0.3 | 2263262 | 464730 | 60058 | 894551 | 9655 | 21944 | 1271726 | 54 | 12 |
| 0.3-0.4 | 1431922 | 292022 | 37535 | 567582 | 5470 | 13316 | 804303 | 34 | 8 |
| 0.4-0.5 | 875030 | 179798 | 22295 | 344562 | 3616 | 8579 | 493690 | 17 | 2 |
| 0.5-0.6 | 460169 | 93416 | 11806 | 179390 | 1662 | 4309 | 262017 | 13 | 1 |
| 0.6-0.7 | 215931 | 44491 | 5551 | 84667 | 805 | 1956 | 122374 | 11 | 0 |
| 0.7-0.8 | 86676 | 17420 | 2345 | 34405 | 328 | 770 | 48718 | 1 | 1 |
| 0.8-0.9 | 26838 | 5113 | 640 | 11862 | 91 | 188 | 13848 | 0 | 0 |
| 0.9-1 | 7356 | 1512 | 170 | 4148 | 65 | 73 | 2883 | 0 | 0 |
| Sum | 20263579 | 4184101 | 562149 | 8146496 | 106313 | 203368 | 11206734 | 858 | 140 |
