# Supplementary figures and images for: Genetics of adaptation in modern chicken
Source: PLoS Genet. 2019 Apr 29;15(4):e1007989. doi: 10.1371/journal.pgen.1007989 (PMC6508745; doi:10.1371/journal.pgen.1007989)

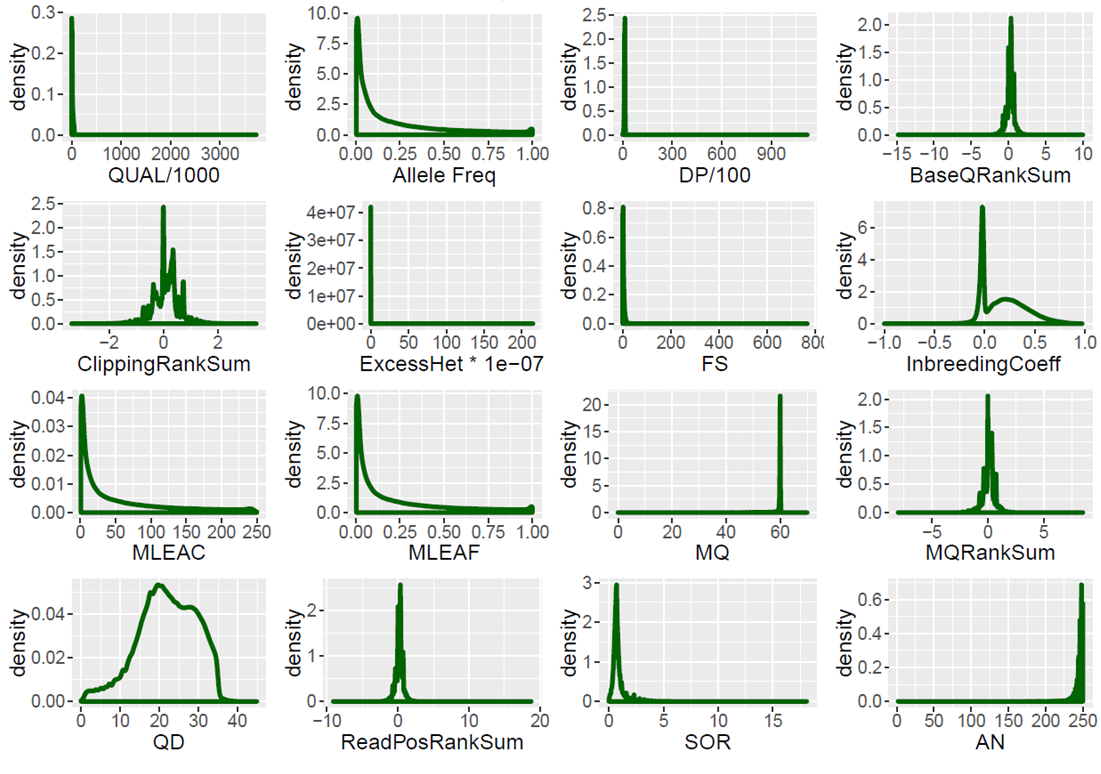

Supplement: S1 Fig — SNPs called according to the best practices workflow using GATK (McKenna et al., 2010). The quality parameters shown from left to right and top to bottom are: Phred Quality Score; Allele Frequency; Depth of Coverage; Base Quality Rank Sum; Clipping Rank Sum; Excess of Heterozygosity; Fisher Strand; Inbreeding Coefficient; Maximum Likelihood Expectation for the Allele counts; Maximum Likelihood Expectation for the Allele Frequency; Mapping Quality; Mapping Quality Rank Sum; Quality by Depth; Z-score from Wilcoxon rank sum test of Alt vs. Ref read position bias; Strand Odds Ratio and Allelic Number in called genotypes. (TIF) [file pgen.1007989.s019.tif]

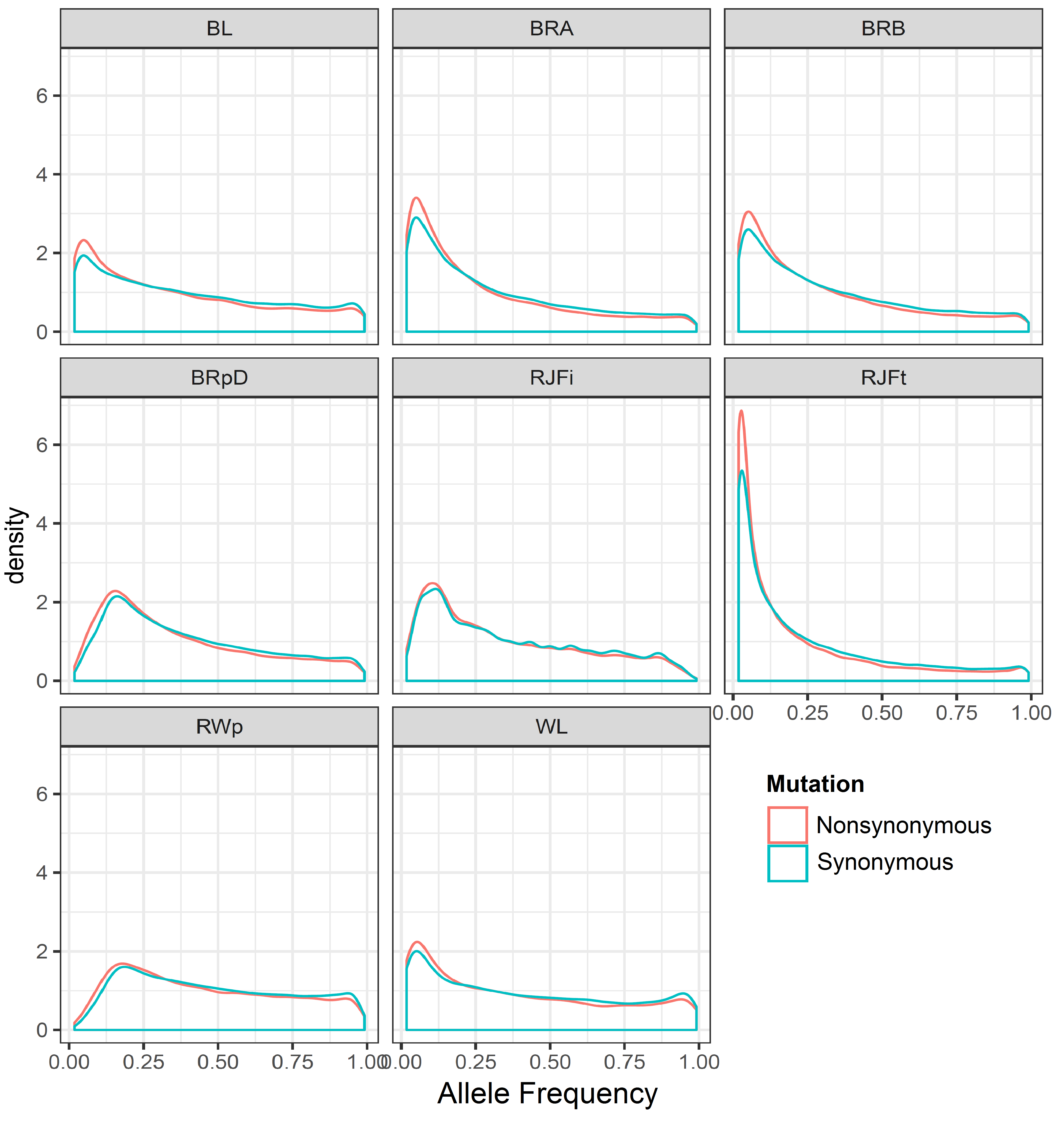

Supplement: S2 Fig — Colored lines depict the distribution of alternative allele for SNPs in different annotation categories. (TIF) [file pgen.1007989.s020.tif]

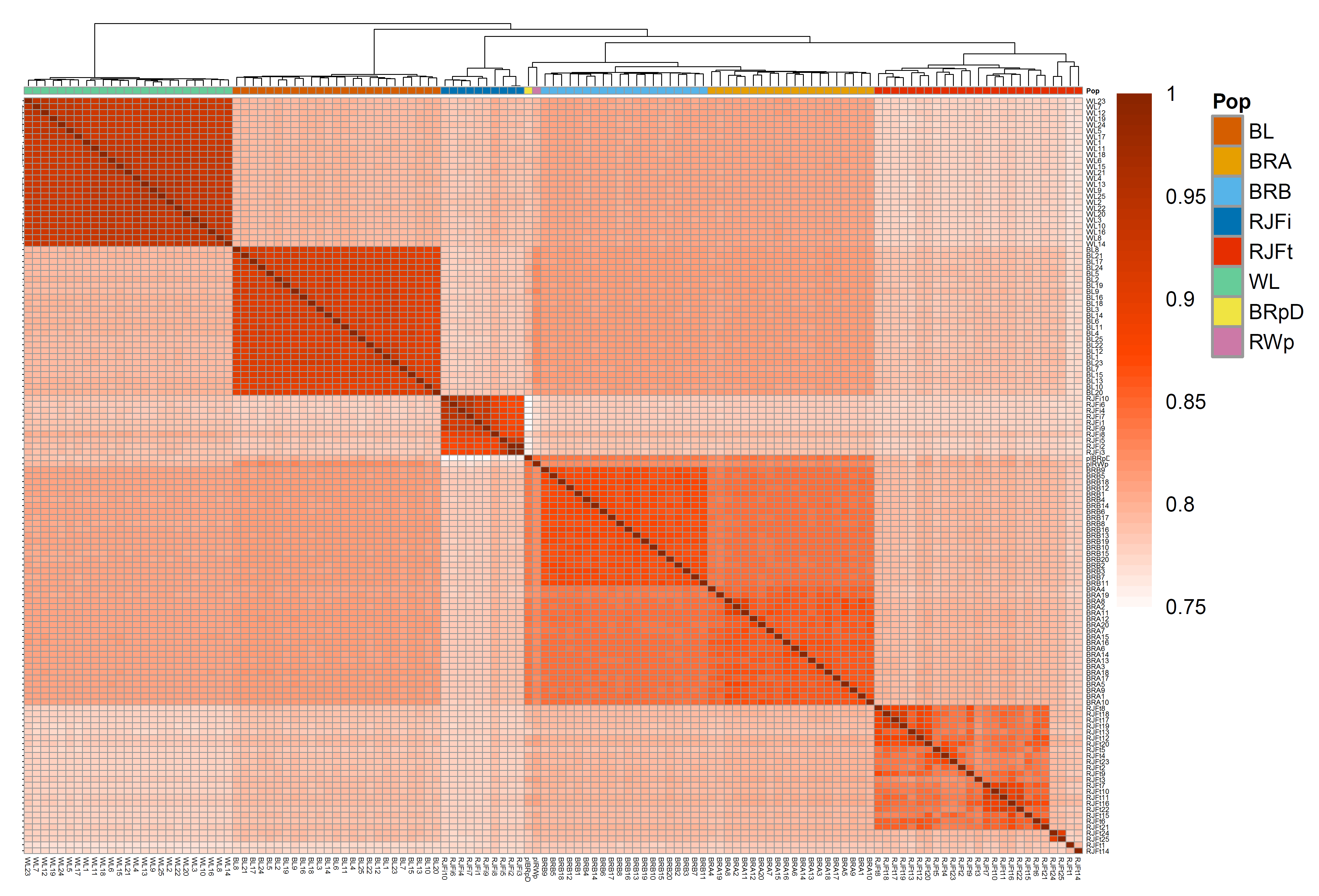

Supplement: S3 Fig — (TIF) [file pgen.1007989.s021.tif]

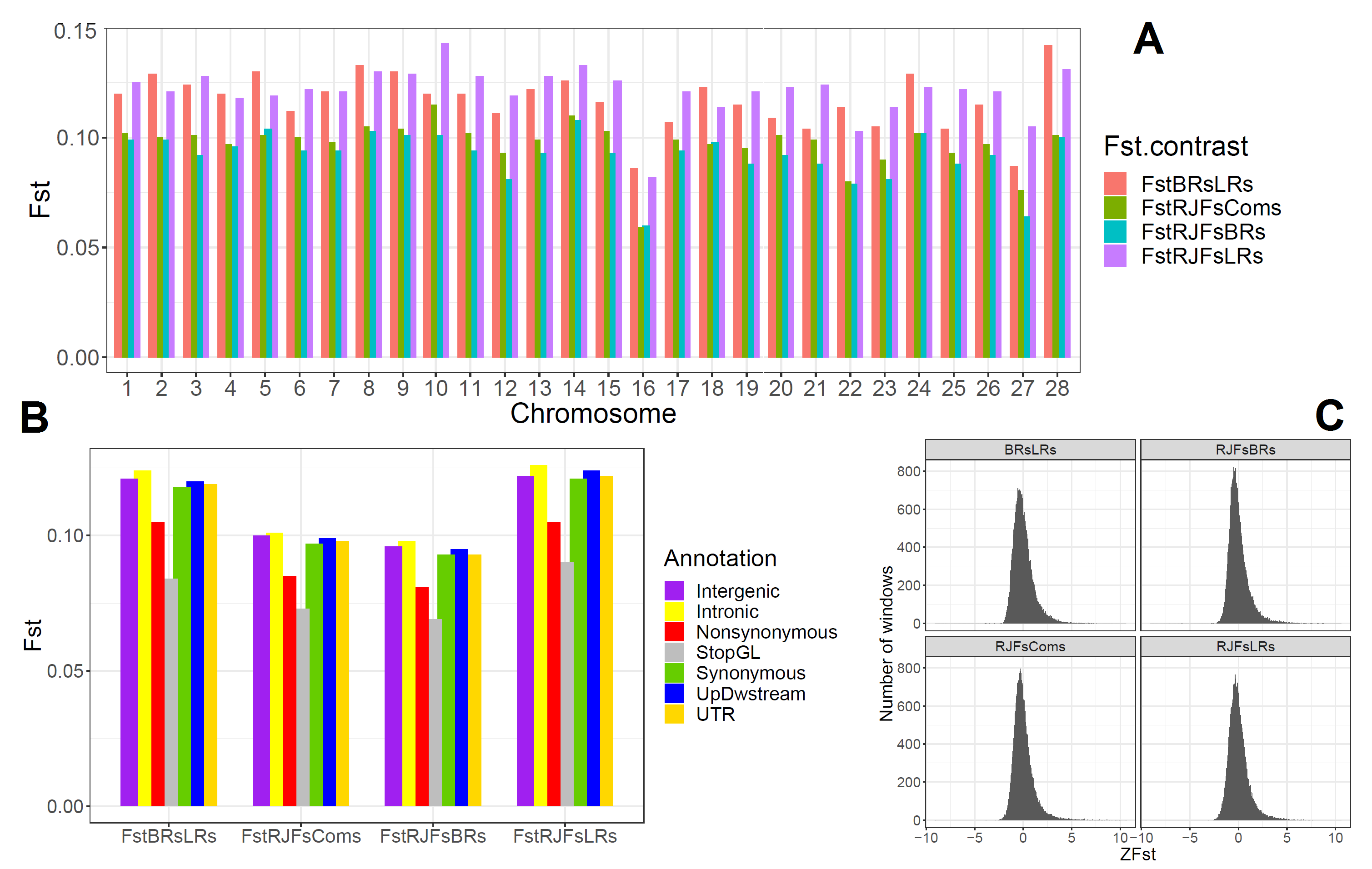

Supplement: S4 Fig — (A) FST among chromosomes in different contrasts of differentiation. The average FST values varied both among chromosomes and between autosomes and chromosome Z. (B) Average FST for different categories of SNPs. Function-altering variants such as stop-gain or loss as well as missense mutations show lower degrees of differentiation than other annotation categories. (C) Distribution of ZFST-scores averaged over 40 kb windows in different contrasts. (TIF) [file pgen.1007989.s022.tif]

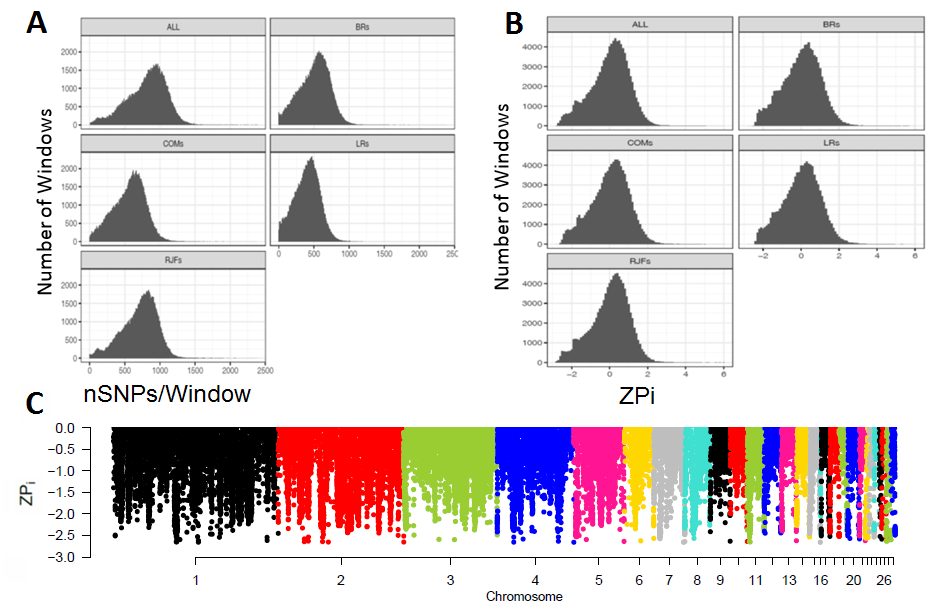

Supplement: S5 Fig — Panel A and B, respectively displays distribution of number of variants and ZPi scores estimated in 40 kb windows in steps of 20 kb in different groups of birds. Panel C provides a schematic representation of the genome-wide nucleotide diversity (ZPi-scores). Nucleotide diversity are estimated only for the six individually sequenced populations. Each dot represents a ZPi-score for a 40 kb window. (TIF) [file pgen.1007989.s023.tif]

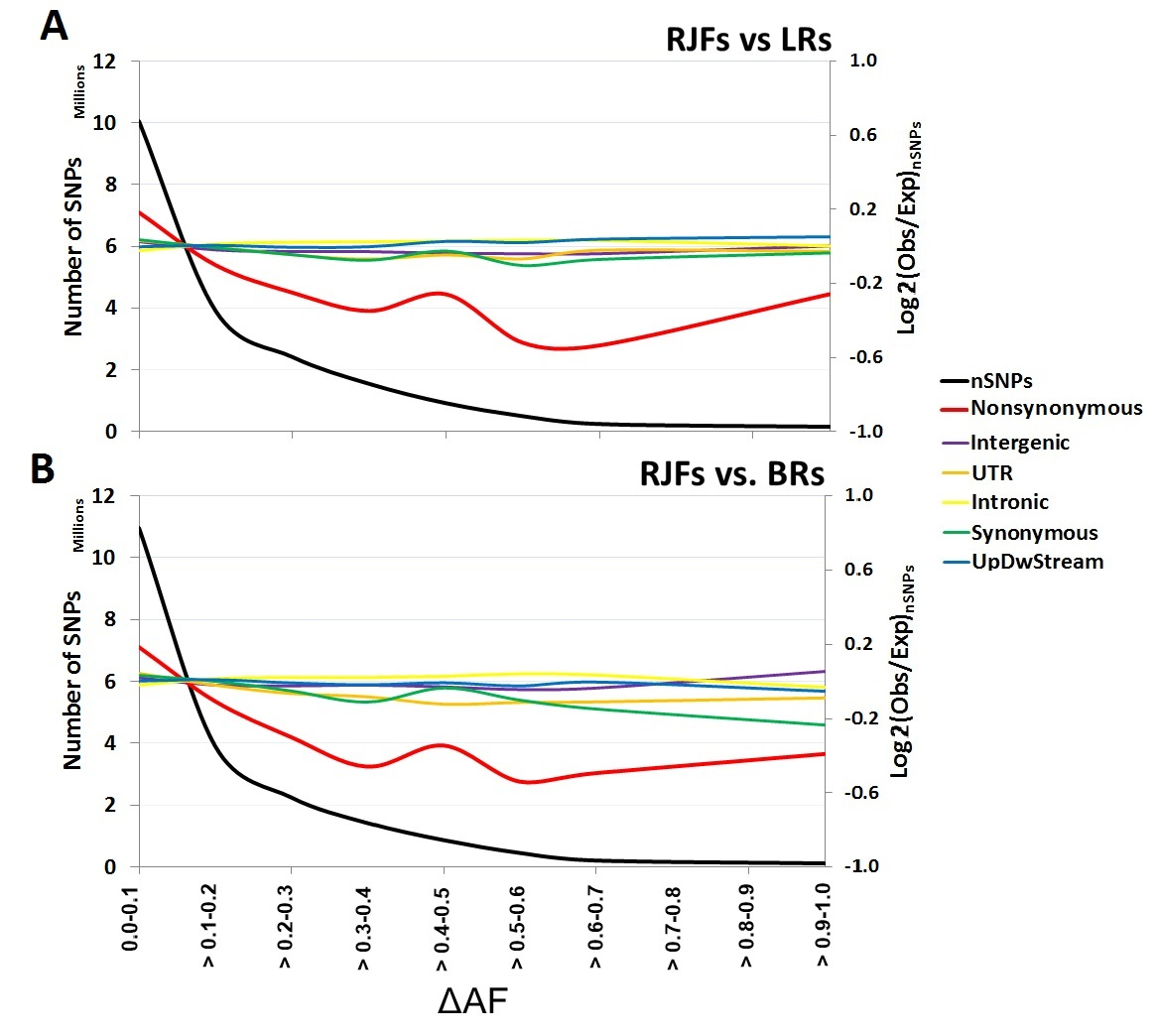

Supplement: S6 Fig — Panel A and B represent the contrasts ‘RJFs vs. LRs’ and ‘RJFs vs. BRs’, respectively. The Y axis represents number of SNPs. The black line represents the total number of SNPs in each ΔAF bin and the colored lines represent log2-fold changes of the observed SNP count for each category in each bin against the expected SNP count. UpDwStream stands for SNPs residing 5 kb up or downstream of genes. (TIF) [file pgen.1007989.s024.tif]
